# Supplementary material for: Impact of night and shift work on metabolic syndrome and its components: a cross-sectional study in an active middle-to-older-aged population-based sample
Source: BMJ Open. 2022 Feb 15;12(2):e053591. doi: 10.1136/bmjopen-2021-053591 (PMC8852754; doi:10.1136/bmjopen-2021-053591)
Supplement: Supplementary data [file bmjopen-2021-053591supp001.pdf]

SUPPLEMENTAL MATERIAL

Impact of night and shift work on metabolic syndrome and its components in an active middle-aged population-based sample

Virginie Bayon,[1,*] Mathieu Berger,[1,*] Geoffroy Solelhac,[1] José Haba-Rubio,[1] Pedro Marques-Vidal,[2] Marie-Pierre Strippoli,[3] Martin Preisig,[3] Damien Leger,[4,5] Raphael Heinzer[1]

[1]Center for Investigation and Research in Sleep (CIRS), Lausanne University Hospital (CHUV) and University of Lausanne, Lausanne, Switzerland; [2]Department of Medicine, Internal Medicine, Lausanne University Hospital (CHUV) and University of Lausanne, Lausanne, Switzerland; [3]Department of Psychiatry, Lausanne University Hospital (CHUV) and University of Lausanne, Lausanne, Switzerland; [4]Université de Paris, VIFASOM (Equipe d'accueil 5330 VIgilance FAtigue SOMmeil et Santé Publique), Paris, France ; [5]APHP, Hôtel Dieu, Centre du Sommeil et de la Vigilance, Paris, France.

Online Data Supplement:

- **1 Supplementary Figure**
- **1 Supplementary Table**

Supplementary Figure 1. Study flowchart

FU: Follow-up

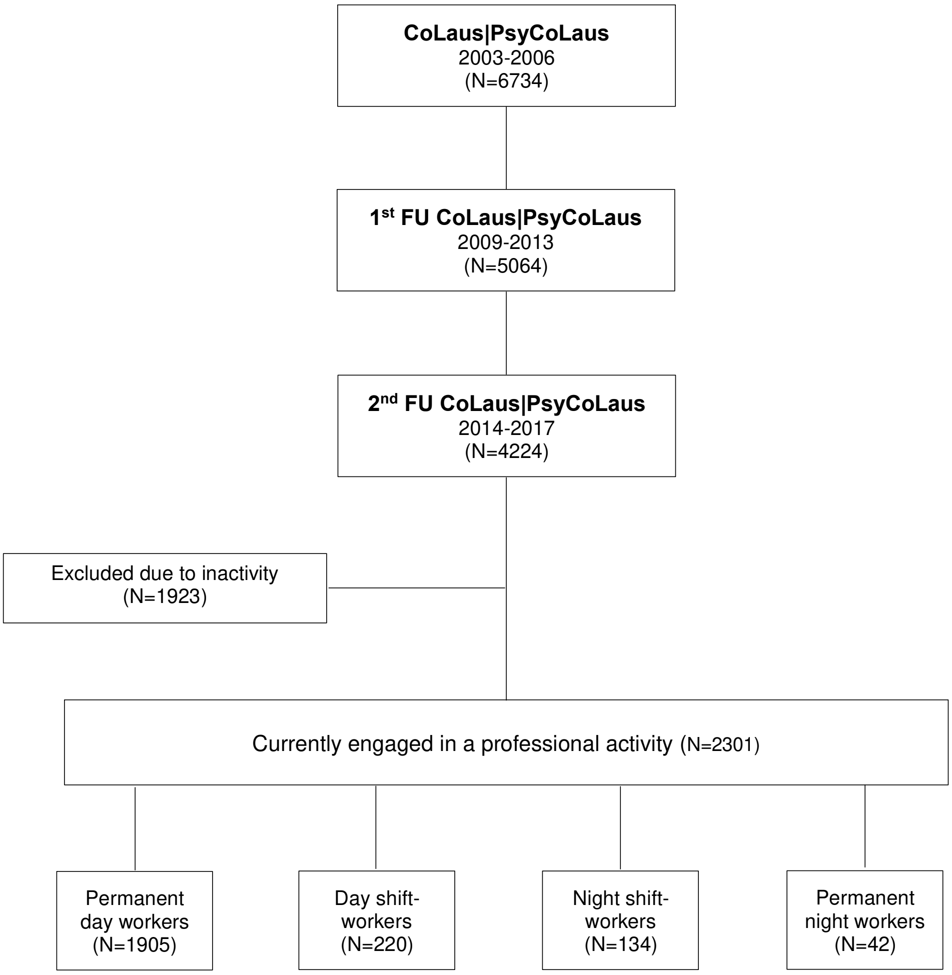

Supplementary Table 1. Association of metabolic syndrome with working schedule

	Crude		Model 1		Model 2	
	**N (%)**	***p*-value**	**OR (95% CI)**	***p*-value**	**OR (95% CI)**	***p*-value**
**Men**	*n* = 1153		*n* = 1059		*n* = 847	
Permanent day workers	Ref	-	Ref	-	Ref	-
Day shift-workers	0.36 (0.18-0.74)	**0.005**	0.33 (0.15-0.69)	**0.004**	0.38 (0.17-0.87)	**0.022**
Night shift-workers	0.78 (0.45-1.34)	0.365	0.71 (0.40-1.26)	0.238	0.67 (0.33-1.36)	0.266
Permanent night workers	6.48 (2.40-17.46)	**<0.001**	6.00 (2.14-16.80)	**0.001**	4.37 (1.33-14.38)	**0.015**
**Women**	*n* = 1148		*n* = 1048		*n* = 798	
Permanent day workers	Ref	-	Ref	-	Ref	-
Day shift-workers	1.17 (0.66-2.05)	0.594	1.15 (0.62-2.12)	0.653	0.47 (0.17-1.24)	0.929
Night shift-workers	1.76 (0.80-3.89)	0.159	1.96 (0.86-4.46)	0.107	1.38 (0.42-4.57)	0.596
Permanent night workers	2.20 (0.81-6.03)	0.124	1.84 (0.60-5.64)	0.289	1.43 (0.28-7.19)	0.668

p-values <0.05 are in bold.

Model 1 was adjusted for age (continuous), age square (continuous) and educational level (middle, low, high). Model 2 was additionally adjusted for weekly alcohol consumption (continuous), smoking status (never, former, current) and daily total energy expenditure.
