# Supplementary material for: Impact of night and shift work on metabolic syndrome and its components: a cross-sectional study in an active middle-to-older-aged population-based sample
Source: BMJ Open. 2022 Feb 15;12(2):e053591. doi: 10.1136/bmjopen-2021-053591 (PMC8852754; doi:10.1136/bmjopen-2021-053591)
Supplement: Author's manuscript [file bmjopen-2021-053591.draft_revisions.pdf]

BMJ Open is committed to open peer review. As part of this commitment we make the peer review history of every article we publish publicly available.

When an article is published we post the peer reviewers' comments and the authors' responses online. We also post the versions of the paper that were used during peer review. These are the versions that the peer review comments apply to.

The versions of the paper that follow are the versions that were submitted during the peer review process. They are not the versions of record or the final published versions. They should not be cited or distributed as the published version of this manuscript.

BMJ Open is an open access journal and the full, final, typeset and author-corrected version of record of the manuscript is available on our site with no access controls, subscription charges or pay-per-view fees (http://bmjopen.bmj.com).

If you have any questions on BMJ Open's open peer review process please email info.bmjopen@bmj.com

BMJ Open

Impact of night and shift work on metabolic syndrome and its components in an active middle-aged population-based sample

Journal:	*BMJ Open*
Manuscript ID	bmjopen-2021-053591
Article Type:	Original research
Date Submitted by the Author:	20-May-2021
Complete List of Authors:	Bayon, Virginie; CHUV) and University of Lausanne<br>Berger, Mathieu; CHUV, Center for Investigation and Research in Sleep<br>Solelhac, Geoffroy; CHUV) and University of Lausanne<br>Haba-Rubio, José; CHUV) and University of Lausanne<br>Marques-Vidal, Pedro; CHUV) and University of Lausanne, Department of Medicine, Internal Medicine<br>Strippoli, Marie-Pierre; CHUV) and University of Lausanne, Department of Psychiatry<br>Preisig, Martin; CHUV) and University of Lausanne, Department of Psychiatry<br>Leger, Damien; Centre du sommeil et de la vigilance, Hôtel Dieu, APHP; Université Paris Descartes, Sorbonne paris Cité, EA 7330 VIFASOM, Sommeil-Vigilance-Fatigue et Santé Publique,<br>Heinzer, Raphael; CHUV) and University of Lausanne
Keywords:	General endocrinology < DIABETES & ENDOCRINOLOGY, EPIDEMIOLOGY, Diabetes & endocrinology < INTERNAL MEDICINE

SCHOLARONE™
Manuscripts

I, the Submitting Author has the right to grant and does grant on behalf of all authors of the Work (as defined in the below author licence), an exclusive licence and/or a non-exclusive licence for contributions from authors who are: i) UK Crown employees; ii) where BMJ has agreed a CC-BY licence shall apply, and/or iii) in accordance with the terms applicable for US Federal Government officers or employees acting as part of their official duties; on a worldwide, perpetual, irrevocable, royalty-free basis to BMJ Publishing Group Ltd ("BMJ") its licensees and where the relevant Journal is co-owned by BMJ to the co-owners of the Journal, to publish the Work in this journal and any other BMJ products and to exploit all rights, as set out in our licence.

The Submitting Author accepts and understands that any supply made under these terms is made by BMJ to the Submitting Author unless you are acting as an employee on behalf of your employer or a postgraduate student of an affiliated institution which is paying any applicable article publishing charge ("APC") for Open Access articles. Where the Submitting Author wishes to make the Work available on an Open Access basis (and intends to pay the relevant APC), the terms of reuse of such Open Access shall be governed by a Creative Commons licence – details of these licences and which Creative Commons licence will apply to this Work are set out in our licence referred to above.

Other than as permitted in any relevant BMJ Author's Self Archiving Policies, I confirm this Work has not been accepted for publication elsewhere, is not being considered for publication elsewhere and does not duplicate material already published. I confirm all authors consent to publication of this Work and authorise the granting of this licence.

For peer review only

Impact of night and shift work on metabolic syndrome and its components in an active middle-aged population-based sample

Short title: Impact of night and shift work

Virginie Bayon, MD[1,*] Mathieu Berger,PhD[1,*] Geoffroy Solelhac,MD[1] José Haba-Rubio,MD[1] Pedro Marques-Vidal,MD[2] Marie-Pierre F. Strippoli,MSc[3] Martin Preisig,MD[3] Damien Leger,MD[4,5] Raphael Heinzer,MD[1]

[1]Center for Investigation and Research in Sleep (CIRS), Lausanne University Hospital (CHUV) and University of Lausanne, Lausanne, Switzerland; [2]Department of Medicine, Internal Medicine, Lausanne University Hospital (CHUV) and University of Lausanne, Lausanne, Switzerland; [3]Department of Psychiatry, Lausanne University Hospital (CHUV) and University of Lausanne, Lausanne, Switzerland; [4]Université de Paris, VIFASOM (Equipe d'accueil 5330 VIgilance FAtigue SOMmeil et Santé Publique), Paris, France ; [5]APHP, Hôtel Dieu, Centre du Sommeil et de la Vigilance, Paris, France

[*]these authors contributed equally to this work

Corresponding Author: Mathieu Berger, PhD, Center for Investigation and Research in Sleep (CIRS), Lausanne University Hospital (CHUV), Rue du Bugnon 46, 1011 Lausanne, Switzerland. Tel: +41 21 314 86 45. E-mail: *mathieuberger@outlook.com*

For peer review only

Total word count (main text only): 3357.

Number of Tables/Figures: 3/1

For peer review only

For peer review only - http://bmjopen.bmj.com/site/about/guidelines.xhtml

ABSTRACT

Objectives To examine the effects of work schedules on metabolic syndrome and its components in active middle-aged workers.

Methods A cross-sectional analysis including active workers from the population-based CoLaus|PsyCoLaus study (Lausanne, Switzerland) was performed. Work schedule was self-reported and defined as follows: permanent day, day shift, night shift, and permanent night work. Associations between work schedule and the risk of metabolic syndrome and its components were analyzed using multivariable-adjusted logistic regressions.

Results A total of 2301 active workers (mean age 56.2 ± 6.9 years, 50.1% women) were included. Of these, 1905 were permanent day workers, 220 were day shift workers, 134 were night shift workers and 42 were permanent night shift workers. There were significant interactions between sex and work schedule for metabolic syndrome, high triglycerides and visceral obesity. Men but not women permanent night workers had a higher prevalence of metabolic syndrome than permanent day workers in multivariable-adjusted analyses (OR 4.45 [95% CI 1.36-14.56]). Analysis of metabolic syndrome subcomponents showed that the association between work schedule and metabolic syndrome in men was mainly driven by visceral obesity (OR 3.35 [95% CI 1.04-10.76]). Conversely, women but not men working in night shift were at increased risk of having high triglycerides compared with permanent day workers (OR 2.92 [95% CI 1.03-8.27]).

Conclusions The risk of metabolic syndrome is higher in men working in permanent night shift compared with permanent day work and this association could be mediated by visceral obesity.

Keywords work schedule, abdominal obesity, risk factors

For peer review only - http://bmjopen.bmj.com/site/about/guidelines.xhtml

For peer review only

For peer review only - http://bmjopen.bmj.com/site/about/guidelines.xhtml

STRENGHS AND LIMITATIONS OF THIS STUDY

- This study evaluated the effects of work schedules on metabolic syndrome and its subcomponent in a general population setting with a precise and extensive assessment of cardio-metabolic phenotypes.
- The association between different shift work schedules and metabolic syndrome was assessed after adjustment for multiple cofounders.
- Because the primary aim of the cohort was not to evaluate the impact of shift work, no precise characterization of workstations and work rhythms (hourly amplitude, direction of rotation, duration of rotations, and duration of exposition) was performed.
- A “healthy worker effect” with a selection of “night shift tolerant” workers cannot be ruled out given the older age of our sample.

For peer review only

For peer review only - http://bmjopen.bmj.com/site/about/guidelines.xhtml

Introduction

Due to economic constraints, efficiency needs or performance objectives, night and shift work (3x8) has become highly prevalent in modern societies. Approximately 18% of all European workers work shifts, and this rate is as high as 35% in some countries[1]. Non-standard working schedules (e.g. shift work, night work) are no longer limited to health and safety workers, but are spread across all industries and services, from manufacturing, to transport, telecommunications and more.

Night and shift work interfere with the physiological circadian rhythm, desynchronizing the biological clock, which can favor systemic inflammation[2]. It has also been shown that night and shift work are associated with reduced and disturbed sleep[3]. Hence, both circadian disruption and short or poor sleep could be mediators explaining the relationship between night or shift work and chronic health conditions, including increased risk of cardiovascular and metabolic disorders[4]. Moreover, several laboratory-controlled studies showed that circadian rhythm desynchronization and sleep restriction have detrimental effects on neuroendocrine, inflammatory and immune functions[5].

The health-related impact of atypical work schedules has thus been a topic of interest for some time[6]. Sleep disturbances, decreased vigilance and increased risk of accidents are among the recognized short-term negative effects of night and shift work[7]. Longer-term health effects have also been described, and include increased risk of cardiovascular and metabolic disorders[8 9]. However, the impact of shift work on metabolic syndrome is not yet completely understood.

Metabolic syndrome combines several interrelated metabolic risk factors associated with all-cause mortality[10]. Subjects with metabolic syndrome have a higher risk of cardiovascular disease mortality and morbidity[11]. Metabolic syndrome definition is

For peer review only - http://bmjopen.bmj.com/site/about/guidelines.xhtml

based on five components: high blood pressure (BP), hyperglycemia, high triglycerides, low high-density lipoprotein (HDL) cholesterol and visceral obesity. A higher prevalence of metabolic syndrome and its components among night and shift workers has previously been suggested in some studies[12 13]. However, the specific effect of shift work and permanent night work remains largely unknown. Moreover, a recent systematic review concluded that there was insufficient evidence regarding the association between shift work and metabolic syndrome when confounding variables are taken into account[14].

Thus, using data of active workers from a population-based study, the aim of the present paper was to assess the association between metabolic syndrome and its components according to four types of work schedules (permanent day, day shift, night shift and permanent night shift work).

Methods

Population CoLaus|PsyCoLaus is a population-based cohort exploring the biological, genetic, and environmental determinants of cardiovascular risk factors, cardiovascular diseases, and mental disorders in the population of Lausanne, Switzerland. The methodological aspects (participant recruitment and follow-up) have been previously reported[15]. Briefly, a simple, non-stratified, random sample of 6,734 subjects from the Lausanne population aged 35-75 years was recruited between 2003 and 2006. The baseline and three follow-up evaluations included physical and psychiatric exams, blood sampling, and self-completed questionnaires. All data analyzed in the present paper were obtained from the second physical follow-up evaluation (n = 4881), which took place between 2014 and 2017. The study was

For peer review only - http://bmjopen.bmj.com/site/about/guidelines.xhtml

approved by the Institutional Ethics Committee of the University of Lausanne (decision reference 33/09) and written inform consent was obtained from all subjects.

Patient and Public Involvement

No patients or public were involved in this study design, conduct or analysis.

Exposure and eligibility criteria Professional activity and working hours were self-reported using the following questions: “Are you currently engaged in a professional activity?”; “What is your usual work schedule?” (day exclusively, rotation with no night work, rotation with night work, night work only). The number of work hours per week was also recorded. Participants not currently engaged in a professional activity were excluded from the present analysis. No other exclusion criteria were applied.

Outcome assessment Metabolic syndrome was defined according to the Joint Interim Statement [16] as the presence of at least three of the following five conditions: high BP (systolic BP ≥130 mm Hg or diastolic BP ≥85 mm Hg or use of antihypertensive medication); visceral obesity (waist circumference ≥88 cm in women or ≥102 cm in men); high triglycerides (≥1.7 mmol/L, or use of fibrates or nicotinic acid); low HDL-cholesterol levels (<1.30 mmol/L in women or <1.03 mmol/L in men, or use of fibrates or nicotinic acid); and high fasting plasma glucose (≥5.6 mmol/L or use of anti-diabetic medication). Blood pressure was measured three times on the left arm after at least a 10-min rest in the seated position. The mean of the last two measures was used. Venous blood samples were drawn after an overnight fast to measure the levels of glucose, HDL cholesterol, low HDL-cholesterol, and triglycerides. Waist circumference was measured twice with a non-stretchable tape over the unclothed abdomen at the mid-point between the lowest rib and the iliac

For peer review only - http://bmjopen.bmj.com/site/about/guidelines.xhtml

crest. Hip circumference was also measured twice at the greater trochanters. For waist and hip, the mean of the two measurements was used and the waist-to-hip ratio (WHR) was calculated.

Covariates The current socio-professional category was self-reported by participants. Sociodemographic (age, sex) and lifestyle (smoking habit, alcohol intake, coffee consumption) data were collected by self-administered questionnaires. Educational level was categorized as *low* (primary), *middle* (apprenticeship or secondary school) or *high* (university). Smoking status was categorized as *never, former* or *current*. Body weight and height were measured with participants standing without shoes in light indoor clothing. Body weight was measured in kilograms to the nearest 0.1 kg using a Seca™ scale (Seca, Hamburg, Germany). Height was measured to the nearest 5 mm using a Seca™ height gauge (Seca, Hamburg, Germany). Body mass index (BMI) was defined as weight (kg)/height^2 (m^2). Obesity was defined as BMI ≥ 30 kg/m^2.

Medication use was coded according to the World Health Organization Anatomical Therapeutic Chemical (ATC) Classification System (http://www.whocc.no/atcddd). Drugs influencing sleep included hypnotics or sedatives (N05C), anxiolytics (N05B) and antipsychotics (N05A). Diabetes was defined as fasting plasma glucose levels ≥7.0 mmol/L or use of antidiabetic medication [17]. Hypertension was defined as systolic BP ≥140 mm Hg and/or diastolic BP ≥90 mm Hg, and/or current use of antihypertensive medication.

Fasting blood sample was collected for various analyses including glucose, total cholesterol, HDL-cholesterol, triglycerides and insulin. The HOMA-IR was calculated as fasting insulin in mIU/L x fasting glucose in mg/dL/405.

For peer review only - http://bmjopen.bmj.com/site/about/guidelines.xhtml

The presence of a current major depressive disorder was retrospectively assigned according to Diagnostic and Statistical Manual of Mental Disorders, Fourth Edition (DSM-IV) criteria with information collected at the second and third psychiatric follow-up evaluation using the French translation of the semi-structured Diagnostic Interview for Genetic Studies (DIGS). Cardiovascular disease was defined as previous stroke, heart attack, coronary artery bypass grafting or percutaneous coronary intervention.

Subjective sleep characteristics were determined using the Pittsburgh Sleep Quality Index (PSQI)[18], the Epworth Sleepiness Scale (ESS)[19], and the Berlin questionnaire for sleep-disordered breathing (SDB)[20]. Sleep quality was assessed with the PSQI and dichotomized into good/poor sleep quality (score ≤5/>5), and excessive daytime sleepiness (EDS) was defined as an ESS score >10). A Berlin score ≥2 was defined as indicating a high risk of SDB.

Dietary intake was evaluated using a validated Food Frequency Questionnaire (FFQ) querying the consumption of 97 different food items including portion size over the previous 4 weeks. The daily total energy intake was obtained as well as the proportion of macronutrients, alcohol and fibers.

Physical activity was evaluated with the physical activity frequency questionnaire (PAFQ)[21]. The questionnaire lists 70 types of physical activity from various domains (e.g. occupational, housework, leisure time, sports, etc.) and participants indicated the number of days in the past week (0–7) and the duration per day (0–10 h, in 15-minute increments) for each activity. Energy expenditure corresponds to the sum of all the energy expenditure over one week divided by 7 to obtain a mean energy expenditure over a 24-hour period. Sedentary status was defined as spending more than 90% of daily energy in activities below moderate and

For peer review only - http://bmjopen.bmj.com/site/about/guidelines.xhtml

high intensity (defined as requiring at least 4 times the basal metabolic rate [BMR]). The percentage of total energy >4 metabolic equivalents (METS) was also calculated to quantify moderate and high intensity physical activity.

Statistical analysis Data distribution was graphically assessed using a normal Q-Q plot. Data were presented as number of participants (%) for categorical variables, mean ± SD for normal distribution, or median and interquartile range for non-normally distributed continuous variables. Univariate analyses of continuous data were performed using one-way ANOVA or Kruskal Wallis test follow by Bonferroni's post-hoc or Tamhane's T2 as appropriate. Categorical variables were analyzed using Chi-square test or Fisher's exact test as appropriate. The associations between working schedules (permanent day, day shift work, night shift work and permanent night work) and metabolic syndrome (and its subcomponents) were determined using logistic regression analysis. Prior to this, the interaction of sex with the metabolic syndrome and each of its subcomponents was tested. In case of significant interaction, results were presented for both men and women, otherwise results were shown for the whole sample. Each cardiometabolic risk factor was first tested in univariate analysis (crude) then in two models with serial adjustment for potential confounders. Model 1 was adjusted for age (continuous), educational level (low, middle, high) and sex (except in case of significant sex*outcome interaction). Model 2: Model 1 plus weekly alcohol consumption (continuous), smoking status (never, former, current) and BMI (normal weight, overweight, obese; except for visceral obesity). Model 3: Model 2 plus daily total energy expenditure (continuous). Box-Tidwell tests were used to check the assumption of linearity for the logit of each covariate. If the assumption was violated, the square of the covariate was used or the

For peer review only - http://bmjopen.bmj.com/site/about/guidelines.xhtml

covariate was transformed into categorical variable. To assess collinearity between covariates, a linear regression analysis including all covariates was performed, and the variance inflation factor (VIF) was calculated. A VIF ≤5 was considered as absence of multi-collinearity. Results from logistic regression are presented as OR values with 95% CI. Permanent day workers were considered as the reference group.

All statistical analyses were performed using IBM SPSS Statistics version 26.0 for Macintosh (IBM Corp). Significant results were considered for a two-sided test with $p < 0.05$.

Results

Population characteristics A total of 2301 participants were engaged in a professional activity at the second follow-up of the CoLaus|PsyCoLaus study. Among them, 1905 worked exclusively during the daytime (permanent day workers), 220 were rotation workers with no night work (day shift workers), 134 were rotation workers with night work (night shift workers) and 42 worked exclusively during the night (permanent night workers) (Supplementary Figure 1).

Table 1 shows the baseline characteristics of the sample according to the four different work schedules. The mean age of the participants was 56.2 ± 6.9 years and half of the sample (50.1%) were women. The proportions of men/women differed significantly according to work schedule: women were more likely to work in day shift and permanent night shift roles, while men were more likely to do night shift work. Mean BMI and waist circumference were significantly higher in night shift workers and permanent night workers compared with permanent day workers and day shift workers (p<0.001). Permanent night shift workers were more likely to smoke than

For peer review only - http://bmjopen.bmj.com/site/about/guidelines.xhtml

other groups, whereas night shift workers were less sedentary than their counterparts. Lipid levels and blood glucose analysis, and sleep parameters in the different work schedule groups are also shown in Table 1.

Prevalence of metabolic syndrome and its components according to work schedules There were significant interactions between sex and work schedule for metabolic syndrome (p=0.009), high triglycerides (p=0.043) and visceral obesity (p=0.047), but not for high BP, high glucose and low HDL-cholesterol.

The prevalence of the metabolic syndrome was almost three times higher in men permanent night workers compared with men permanent day workers; a similar trend was found for the prevalence of visceral obesity and low HDL-cholesterol (Table 2). The prevalence of high glucose level in night shift workers and permanent night workers was nearly double that in permanent day workers (Table 2).

Association between metabolic syndrome and work schedules by patient sex

Compared to men permanent day workers, permanent night workers showed a higher risk of metabolic syndrome in univariate analysis (OR 6.48 [95% CI 2.40-17.46]; Supplementary Table 1). This significant association persisted after adjustment for age, educational level, alcohol consumption, smoking status and daily total energy expenditure (OR 4.45 [95% CI 1.36-14.56]) (Figure 1). Conversely, the risk of metabolic syndrome in day shift-workers was lower than that in permanent day workers in crude analysis (OR 0.36 [95% CI 0.18-0.74]), and after adjustment in models 1 and 2 and 3 (Supplementary Table 1). No significant association between work schedule and metabolic syndrome was found for women.

For peer review only - http://bmjopen.bmj.com/site/about/guidelines.xhtml

Association of each component of metabolic syndrome with work schedule

In men, the risk of visceral obesity in permanent night workers was significantly higher than that in permanent day workers, including after adjustment for covariates (Table 3). Moreover, the risk of elevated triglyceride levels in permanent night workers was increased in the crude analysis and after adjustment for age, educational level, alcohol consumption, smoking status and BMI (model 2), but was no longer significant in the fully adjusted model 3 (Table 3).

In women, night shift-workers showed a higher risk of elevated triglyceride levels, which persisted after multiple adjustments (Table 3).

Discussion

In our middle-aged active general population sample, we found differential associations between permanent night work and the risk of metabolic syndrome for men and women. Indeed, permanent night work was only associated with a higher risk of metabolic syndrome in men but not in women. This association could be mediated by a higher risk of visceral obesity in men. The increased risk of metabolic syndrome is in line with previous studies[22]. Some studies even showed that the risk for the development of metabolic syndrome and each of its components gradually and independently increases with accumulated years of shift work[23]. Contrary to other studies, we found no association between permanent night work or night shift-work and metabolic syndrome in women[24 25]. In contrast to the findings on the metabolic syndrome as a whole, for the triglycerides component we found an increased risk of elevated concentrations among shift workers in women but not in men. This supports previous evidence from Karlsson *et al.* who also reported an elevated triglyceride level among shift workers in 60-year-old women[26].

For peer review only - http://bmjopen.bmj.com/site/about/guidelines.xhtml

While the mechanisms underlying the observed increased risk of metabolic syndrome in shift or night workers have not been fully elucidated, several explanatory hypotheses can be proposed. Firstly, sleep duration has been suggested to play a key role in the development of metabolic syndrome. A previous meta-analysis found that short sleep duration was significantly associated with a 27% increase in risk of metabolic syndrome whereas long sleep duration was not[27]. Similar results were found in both men and women. In our study, self-reported sleep duration did not differ between the different groups of workers and therefore does not explain the increased risk of metabolic syndrome observed in permanent night workers among men. However, we cannot rule out that our findings might have been different if objective sleep duration measures were used because objective and subjective sleep duration can differ significantly. Unfortunately, objective sleep assessment could not be included in our analysis. Moreover, sleep fragmentation or an alteration of sleep structure due to irregular sleep schedule or circadian rhythm misalignment in night workers cannot be excluded and could be a possible explanation for the increased risk of metabolic syndrome[28 29].

Secondly, dietary habits could contribute to development of the metabolic syndrome in night or shift workers, but available studies on this subject are scarce. A cross-sectional study comparing 98 rotating shift workers to 100 regular day workers demonstrated that total energy intake and contributions of macronutrients did not differ between the two groups, except for saturated lipids (+10% in shift workers)[30]. However, meal distribution was different in the two groups. Similar to other studies[31 32], we failed to demonstrate a difference in food intake and macronutrients components between night shift workers or permanent night workers compared with permanent day workers. Available data from our study mean that, unfortunately, we

For peer review only - http://bmjopen.bmj.com/site/about/guidelines.xhtml

cannot rule out the possibility that night shift workers may have had a different circadian distribution of food intake rather than an increase in total daily intake[33].

Thirdly, circadian rhythm desynchronization could be a major contributor to the increased risk of metabolic syndrome among night and shift workers. Still, the underlying pathophysiological mechanisms of this association remain poorly understood. Some animal studies suggested that reduced melatonin production, due to circadian rhythm disruption, could be associated with a higher rate of metabolic syndrome[34]. Furthermore, Fonken *et al.* hypothesized that exposure to light at night altered circadian organization and affected metabolic parameters in mice[35]. Their results emphasized that even weak night lighting (5 lux) is sufficient to desynchronize food consumption and physical activity rhythms, which could explain the observed metabolic disorders[34]. In humans, Corbalan-Tutau *et al.* reported a reduced daily amplitude in melatonin and cortisol circadian patterns associated with metabolic disturbances in women[36]. Unfortunately, we did not measure melatonin and cortisol to confirm these findings in our sample.

With regard to physical activity, we surprisingly found that night shift workers were more active than day shift workers and permanent day workers. This may be due to greater opportunities to perform a physical activity compared with other diurnal workers or to more physically active work among night shift workers, although this should be interpreted with caution due to limited agreement between estimates of activity obtained by PAFQ and those obtained from accelerometers[37].

Finally, the higher risk of metabolic syndrome we observed in night shift workers may be explained by a vitamin D deficiency[38]. It has been shown that high levels of vitamin D among middle-aged and elderly populations are associated with a substantial decrease in cardiovascular disease, type 2 diabetes and metabolic

For peer review only

syndrome[39]. Although we did not measure the vitamin D levels in our different groups of workers, we can hypothesize that permanent night workers have lower exposure to sunlight and may therefore be at higher risk of vitamin D deficiency.

In our study, among the components of the metabolic syndrome, an elevated risk of visceral obesity was found in men permanent night workers. This finding is consistent with a recent meta-analysis which found that shift workers had a higher frequency of abdominal obesity than other obesity types and permanent night workers demonstrated a 29% higher risk of central obesity than rotating shift workers[40].

The main strength of the present study is its large population-based sample with a precise and extensive assessment of cardio-metabolic phenotypes. Indeed, previous studies were mainly performed in specific populations of workers or in particular sectors of activity, such as public health and emergency, which limit the generalizability to other types of shift or night work. In addition, most studies have assessed the risk of metabolic syndrome in shift workers compared with day workers, but few studies have differentiated between shift workers, permanent night workers and shift workers with and without night work.

There are also some limitations that need to be mentioned. First, because the primary aim of the CoLaus|PsyCoLaus study was not to evaluate the impact of shift work, no precise characterization of workstations and work rhythms (hourly amplitude, direction of rotation, duration of rotations, and duration of exposition) was performed. Second, a “healthy worker effect” with a selection of “night shift tolerant” workers cannot be ruled out given the older age of our sample. Third, there were some missing data on self-reported sleep habits and diet parameters and, despite the use of validated questionnaires, declaration bias remains possible. Similarly, only

For peer review only - http://bmjopen.bmj.com/site/about/guidelines.xhtml

self-reported physical activity was assessed in this study and it would have been interesting to have objective measures of physical activity and sleep to more accurately investigate their influence.

CONCLUSION

our study demonstrates that only men permanent night workers appear to be at increased risk of metabolic syndrome compared with permanent day workers, and this association persisted after adjustment for sociodemographic confounders and daily total energy expenditure. From a clinical point of view, we advise monitor of not only BMI but also visceral obesity, particularly in men permanent night workers. Further studies are needed to elucidate the underline mechanisms.

For peer review only

ARTICLE INFORMATION

Acknowledgments

The authors thank Prof. Mehdi Tafti, Prof. Vincent Mooser, Daniela Andries and Nadia Tobback for their important contribution to the HypnoLaus and CoLaus|PsyCoLaus Cohorts, the Lausanne population who volunteered to participate in the CoLaus|PsyCoLaus and HypnoLaus studies, as well as the whole team of CoLaus|PsyCoLaus.

Funding

The HypnoLaus and the CoLaus/ PsyCoLaus studies were and are supported by research grants from GlaxoSmithKline ('not applicable'), the Faculty of Biology and Medicine of Lausanne ('not applicable'), the Swiss National Science Foundation (grants 3200B0-105993, 3200B0-118308, 33CSCO-122661, 33CS30-139468 and 33CS30- 148401), Leenaards Foundation ('not applicable'), and Vaud Pulmonary League ('not applicable'). The contents of this research are solely the responsibility of the authors.

Author Disclosures

The authors have no conflicts of interest to declare.

Contributors

VB, MB and RH designed the study. MB performed the statistical analysis. VB, MB and RH wrote the first draft of the manuscript. All authors interpreted the data, critically reviewed the manuscript and approved the final version. VB is the guarantor

For peer review only - http://bmjopen.bmj.com/site/about/guidelines.xhtml

of this work and, as such, had full access to all the data in the study and takes responsibility for the integrity of the data and the accuracy of the data analysis.

Data availability statement

Data may be obtained from a third party and are not publicly available. All data relevant to the study are included in the article or uploaded as supplementary information.

References

1 Nappo N. Is there an association between working conditions and health? An analysis of the Sixth European Working Conditions Survey data. *PLoS One* 2019;14:e0211294.

2 Puttonen S, Viitasalo K, Harma M. Effect of shiftwork on systemic markers of inflammation. *Chronobiol Int* 2011;28:528-35.

3 Sallinen M, Kecklund G. Shift work, sleep, and sleepiness - differences between shift schedules and systems. *Scand J Work Environ Health* 2010;36:121-33.

4 Knutson KL, Spiegel K, Penev P, Van Cauter E. The metabolic consequences of sleep deprivation. *Sleep Med Rev* 2007;11:163-78.

5 Faraut B, Bayon V, Leger D. Neuroendocrine, immune and oxidative stress in shift workers. *Sleep Med Rev* 2013;17:433-44.

6 Kecklund G, Axelsson J. Health consequences of shift work and insufficient sleep. *BMJ* 2016;355:i5210.

7 Drake CL, Roehrs T, Richardson G, Walsh JK, Roth T. Shift work sleep disorder: prevalence and consequences beyond that of symptomatic day workers. *Sleep* 2004;27:1453-62.

For peer review only

8 Morikawa Y, Nakagawa H, Miura K et al. Effect of shift work on body mass index and metabolic parameters. *Scand J Work Environ Health* 2007;33:45-50.

9 Wang D, Ruan W, Chen Z, Peng Y, Li W. Shift work and risk of cardiovascular disease morbidity and mortality: A dose-response meta-analysis of cohort studies. *Eur J Prev Cardiol* 2018;25:1293-1302.

10 Wu SH, Liu Z, Ho SC. Metabolic syndrome and all-cause mortality: a meta-analysis of prospective cohort studies. *Eur J Epidemiol* 2010;25:375-84.

11 Galassi A, Reynolds K, He J. Metabolic syndrome and risk of cardiovascular disease: a meta-analysis. *Am J Med* 2006;119:812-9.

12 Karlsson B, Knutsson A, Lindahl B. Is there an association between shift work and having a metabolic syndrome? Results from a population based study of 27,485 people. *Occup Environ Med* 2001;58:747-52.

13 Violanti JM, Burchfiel CM, Hartley TA et al. Atypical work hours and metabolic syndrome among police officers. *Arch Environ Occup Health* 2009;64:194-201.

14 Canuto R, Garcez AS, Olinto MT. Metabolic syndrome and shift work: a systematic review. *Sleep Med Rev* 2013;17:425-31.

15 Firmann M, Mayor V, Vidal PM et al. The CoLaus study: a population-based study to investigate the epidemiology and genetic determinants of cardiovascular risk factors and metabolic syndrome. *BMC Cardiovasc Disord* 2008;8:6.

16 Alberti KG, Eckel RH, Grundy SM et al. Harmonizing the metabolic syndrome: a joint interim statement of the International Diabetes Federation Task Force on Epidemiology and Prevention; National Heart, Lung, and Blood Institute; American Heart Association; World Heart Federation; International Atherosclerosis Society; and International Association for the Study of Obesity. *Circulation* 2009;120:1640-5.

For peer review only

17 American Diabetes A. Diagnosis and classification of diabetes mellitus. *Diabetes Care* 2014;37 Suppl 1:S81-90.

18 Buysse DJ, Reynolds CF, 3rd, Monk TH, Berman SR, Kupfer DJ. The Pittsburgh Sleep Quality Index: a new instrument for psychiatric practice and research. *Psychiatry Res* 1989;28:193-213.

19 Johns MW. A new method for measuring daytime sleepiness: the Epworth sleepiness scale. *Sleep* 1991;14:540-5.

20 Netzer NC, Stoohs RA, Netzer CM, Clark K, Strohl KP. Using the Berlin Questionnaire to identify patients at risk for the sleep apnea syndrome. *Ann Intern Med* 1999;131:485-91.

21 Bernstein M, Sloutskis D, Kumanyika S, Sparti A, Schutz Y, Morabia A. Data-based approach for developing a physical activity frequency questionnaire. *Am J Epidemiol* 1998;147:147-54.

22 Wang F, Zhang L, Zhang Y et al. Meta-analysis on night shift work and risk of metabolic syndrome. *Obes Rev* 2014;15:709-20.

23 De Bacquer D, Van Risseghem M, Clays E, Kittel F, De Backer G, Braeckman L. Rotating shift work and the metabolic syndrome: a prospective study. *Int J Epidemiol* 2009;38:848-54.

24 Lim YC, Hoe VCW, Darus A, Bhoo-Pathy N. Association between night-shift work, sleep quality and metabolic syndrome. *Occup Environ Med* 2018;75:716-723.

25 Guo Y, Rong Y, Huang X et al. Shift work and the relationship with metabolic syndrome in Chinese aged workers. *PLoS One* 2015;10:e0120632.

26 Karlsson B, Knutsson A, Lindahl B. Is there an association between shift work and having a metabolic syndrome? Results from a population based study of 27,485 people. *Occupational and Environmental Medicine* 2001;58:747-752.

For peer review only - http://bmjopen.bmj.com/site/about/guidelines.xhtml

27 Xi B, He D, Zhang M, Xue J, Zhou D. Short sleep duration predicts risk of metabolic syndrome: a systematic review and meta-analysis. *Sleep Med Rev* 2014;18:293-7.

28 Stamatakis KA, Punjabi NM. Effects of sleep fragmentation on glucose metabolism in normal subjects. *Chest* 2010;137:95-101.

29 Tasali E, Leproult R, Ehrmann DA, Van Cauter E. Slow-wave sleep and the risk of type 2 diabetes in humans. *Proc Natl Acad Sci U S A* 2008;105:1044-9.

30 Esquirol Y, Bongard V, Mabile L, Jonnier B, Soulat JM, Perret B. Shift work and metabolic syndrome: respective impacts of job strain, physical activity, and dietary rhythms. *Chronobiol Int* 2009;26:544-59.

31 de Assis MA, Kupek E, Nahas MV, Bellisle F. Food intake and circadian rhythms in shift workers with a high workload. *Appetite* 2003;40:175-83.

32 Morikawa Y, Miura K, Sasaki S et al. Evaluation of the effects of shift work on nutrient intake: a cross-sectional study. *J Occup Health* 2008;50:270-8.

33 Molzof HE, Wirth MD, Burch JB et al. The impact of meal timing on cardiometabolic syndrome indicators in shift workers. *Chronobiol Int* 2017;34:337-348.

34 Fonken LK, Workman JL, Walton JC et al. Light at night increases body mass by shifting the time of food intake. *Proc Natl Acad Sci U S A* 2010;107:18664-9.

35 Fonken LK, Nelson RJ. The effects of light at night on circadian clocks and metabolism. *Endocr Rev* 2014;35:648-70.

36 Corbalan-Tutau D, Madrid JA, Nicolas F, Garaulet M. Daily profile in two circadian markers "melatonin and cortisol" and associations with metabolic syndrome components. *Physiol Behav* 2014;123:231-5.

37 Verhoog S, Gubelmann C, Guessous I, Bano A, Franco OH, Marques-Vidal P. Comparison of the Physical Activity Frequency Questionnaire (PAFQ) with

For peer review only

accelerometry in a middle-aged and elderly population: The CoLaus study. *Maturitas* 2019;129:68-75.

38 Ju SY, Jeong HS, Kim DH. Blood vitamin D status and metabolic syndrome in the general adult population: a dose-response meta-analysis. *J Clin Endocrinol Metab* 2014;99:1053-63.

39 Parker J, Hashmi O, Dutton D et al. Levels of vitamin D and cardiometabolic disorders: systematic review and meta-analysis. *Maturitas* 2010;65:225-36.

40 Sun M, Feng W, Wang F et al. Meta-analysis on shift work and risks of specific obesity types. *Obes Rev* 2018;19:28-40.

For peer review only

For peer review only - http://bmjopen.bmj.com/site/about/guidelines.xhtml

Table 1. Baseline characteristics according to work schedules

	Permanent day workers (*n* = 1905)	Day shift workers (*n* = 220)	Night shift workers (*n* = 134)	Permanent night workers (*n* = 42)	*p*-value	N Total
**Demographics**						
Age (years)	55.0 (50.0–60.0)	55.0 (50.5–59.5)	54.5 (50.4–58.6)	53.0 (48.8–57.2)	0.070	2275
Men, n (%)	958 (50.3)	89 (40.5) [a]	88 (65.7) [a]	18 (42.9)	**<0.001**	2301
Educational level, n (%)					**<0.001**	2300
Low	791 (41.5)	112 (50.9)	71 (53.0)	30 (71.4) [a]		
Middle	522 (27.4)	65 (29.5)	40 (29.9)	10 (23.8)		
High	591 (31.0) [a]	43 (19.5)	23 (17.2)	2 (4.8)		
**Anthropometrics**						
BMI (kg/m^2)	25.4 (22.6–28.5)	25.5 (23.1–27.6)	26.0 (23.2–30.0) [b]	27.9 (25.4–31.3) [b,c]	**<0.001**	2228
Waist circumference (cm)	89.5 (81.0–98.5)	89.0 (81.4–96.0)	93.0 (84.3–102.0) [b,c]	95.0 (85.3–109.0) [b,c]	**<0.001**	2227
Waist to hip ratio	0.88 ± 0.09	0.87 ± 0.08	0.90 ± 0.09 [b,c]	0.90 ± 0.09	**0.013**	2227
**Risk factors**						
Metabolic syndrome, n (%)	327 (17.2)	25 (11.4)	25 (18.7)	17 (40.5)[a]	**<0.001**	2301
Number of metabolic risk factors†	1 (0–2)	1 (0–2)	1 (0–2)	2 (1–3) [b,c]	**0.006**	2301
Current major depressive disorder, n (%)	115 (7.9)	19 (11.7)	10 (9.6)	4 (12.1)	0.319	1756
Hypertension, n (%)	653 (34.9)	80 (36.7)	45 (33.6)	12 (28.6)	0.764	2263

For peer review only - http://bmjopen.bmj.com/site/about/guidelines.xhtml

Diabetes, n (%)	100 (5.4)	8 (3.7)	18 (13.6) [a]	4 (9.5)	**<0.001**	2231
Dyslipidemia, n (%)	413 (22.5)	51 (23.6)	29 (22.1)	16 (38.1)	0.123	2226
Sleep drugs, n (%)	109 (5.7)	14 (6.4)	4 (3.0)	4 (9.5)	0.367	2301
Cardiovascular disease, n (%)[††]	55 (2.9)	12 (5.5)	5 (3.7)	3 (7.1)	0.102	2291
**Lifestyle factors**						
Smoking status, n (%)					**0.011**	2246
Never	771 (41.4)	81 (38.4)	51 (39.2)	14 (34.1)		
Former	689 (37.0)	86 (40.8)	57 (43.8)	9 (22.0) [a]		
Curent	404 (21.7)	44 (20.9)	22 (16.9)	18 (43.9) [a]		
Alcohol (units/week)	4 (1–9)	3 (0–7)	3 (0–7)	2 (0–6)	**0.010**	2162
Coffee consumption, n (%)					0.961	2222
None	186 (10.1)	23 (11.0)	13 (10.2)	4 (9.8)		
1-3 cups	1154 (62.6)	134 (64.1)	78 (60.9)	28 (68.3)		
≥4 cups	504 (27.3)	52 (24.9)	37 (28.9)	9 (22.0)		
Total energy intake (Kcals/day)	1756 ± 664	1761 ± 654	1828 ± 719	1853 ± 619	0.603	1996
Physical activity						
Total energy expenditure (Kcals/day)	2656 (2297–3076)	2698 (2336–3046)	3118 (2735–3578) [b,c]	2663 (2356–3164)	**<0.001**	1828
Activity ≥4 MET (% total activity)	10.1 (1.9–18.4)	8.8 (1.2–20.2)	14.4 (4.9–25.3) [b]	6.5 (0.3–16.1)	**0.005**	1828
Sedentary status, n (%)	758 (49.2)	92 (55.1)	32 (34.4) [a]	16 (57.1)	**0.011**	1828

For peer review only - http://bmjopen.bmj.com/site/about/guidelines.xhtml

**Blood analysis**						
Total cholesterol (mmol/L)	5.3 ± 0.9	5.4 ± 0.9	5.3 ± 0.9	5.4 ± 1.0	0.928	2226
HDL cholesterol (mmol/L)	1.5 (1.2–1.9)	1.6 (1.3–1.9)	1.4 (1.2–1.8)	1.4 (1.1–1.7)	**0.013**	2226
LDL cholesterol (mmol/L)	3.2 ± 0.8	3.2 ± 0.8	3.2 ± 0.8	3.2 ± 0.9	0.958	2226
Triglycerides (mmol/L)	1.0 (0.8–1.4)	1.0 (0.8–1.5)	1.1 (0.8–1.5)	1.2 (0.9–1.8)	0.278	2226
Fasting glucose (mmol/L)	5.2 (4.9–5.5)	5.1 (4.8–5.5)	5.2 (4.9–5.8)	5.5 (5–5.9)	**0.026**	2226
Insulin (microIU/mL)	7 (4.8–10.6)	7.2 (4.6–10.9)	7.3 (5.0–11.7)	8.8 (6.5–12.9)	**0.027**	2218
HOMA-IR	1.6 (1.1–2.6)	1.6 (1.0–2.6)	1.7 (1.2–3.2)	2.1 (1.5–3.4)	**0.012**	2218
**Sleep & vigilance**						
Epworth Sleepiness Scale score	6 (4–8)	5 (3–8)	6 (4–9)	5 (3–8)	0.623	1786
Excessive daytime sleepiness, n (%)‡	182 (12.1)	20 (12.6)	14 (14.0)	3 (11.1)	0.950	1786
Poor sleep quality, n (%)‡‡	415 (31.5)	46 (37.4)	27 (32.1)	7 (35.0)	0.600	1542
High risk of SDB, n (%)§	321 (21.3)	34 (21.0)	29 (28.4)	8 (27.6)	0.323	1800
Self-reported total sleep time (h)	6.9 ± 1.0	6.8 ± 0.9	6.9 ± 1.0	7.1 ± 1.3	0.507	1542
**Work characteristics**						
Number of working hours/week	38.0 ± 14.7	38.7 ± 15.2	43.1 ± 18.1	38.0 ± 15.2	0.260	2285
Work time, n (%)					0.397	2258
Full-time	1569 (83.8)	181 (84.6)	111 (86.0)	39 (92.9)		
<50%	304 (16.2)	33 (15.4)	18 (14.0)	3 (7.1)		

For peer review only

Example of physical intensity at work, n (%)					**<0.001**	2135
Sedentary (sitting/driving)	1409 (79.5)	105 (51.2)	66 (55.0)	14 (37.8)		
Pushing wheelbarrow	283 (16.0)	81 (39.5)	40 (33.3)	16 (43.2)		
Unloading a truck without assist.	81 (4.6)	19 (9.3)	14 (11.7)	7 (18.9)		

Data are presented as mean ± SD or median and interquartile range for continuous variables and number of participants (%) for categorical variables. Continuous data were analyzed with one-way ANOVA or Kruskal Wallis test follow by Bonferroni's post-hoc or Tamhane's T2 as appropriate. Categorical variables were analyzed using Chi-square test. P-value < 0.05 are shown in bold.

[a] adjusted residual >│ 2│; [b] statistically different from "day only"; [c] statistically different from "shift work without night".

For peer review only

For peer review only - http://bmjopen.bmj.com/site/about/guidelines.xhtml

Table 2. Prevalence of metabolic syndrome and its subcomponents according to work schedule

	**Permanent day workers (*n* = 1905)**	**Day shift workers (*n* = 220)**	**Night shift workers (*n* = 134)**	**Permanent night workers (*n* = 42)**	***p*-value**
Metabolic syndrome					
Men	226 (23.6)	9 (10.1)	17 (19.3)	12 (66.7)	**<0.001**
Women	101 (10.7)	16 (12.2)	8 (17.4)	5 (20.8)	0.225
High BP	826 (43.4)	91 (41.4)	64 (47.8)	23 (54.8)	0.313
High glucose	472 (24.8)	50 (22.7)	47 (35.1)	16 (38.1)	**0.010**
High triglycerides					
Men	243 (26.2)	25 (29.1)	18 (21.2)	11 (61.1)	**0.006**
Women	86 (9.5)	16 (12.3)	9 (19.6)	3 (12.5)	0.183
Low HDL-cholesterol	201 (10.9)	19 (8.8)	10 (7.6)	9 (21.4)	0.064
Visceral obesity					
Men	220 (23.7)	16 (18.6)	23 (26.7)	11 (61.1)	**0.002**
Women	302 (33.3)	55 (42.3)	21 (45.7)	11 (45.8)	0.051

Data are presented as n (%).

Where there was an interaction of outcome*sex, results are presented separately for men and women, otherwise for the whole cohort.

For peer review only - http://bmjopen.bmj.com/site/about/guidelines.xhtml

Table 3. Association of each component of the metabolic syndrome with work schedule

	Crude		Model 1		Model 2		Model 3	
	**OR (95% CI)**	***p*-value**	**OR (95% CI)**	***p*-value**	**OR (95% CI)**	***p*-value**	**OR (95% CI)**	***p*-value**
**High BP**	*n* = 2301		*n* = 2274		*n = 2066*		*n* = 1731	
Permanent day workers	Ref	-	Ref	-	Ref	-	Ref	-
Day shift-workers	0.92 (0.69-1.22)	0.572	1.05 (0.78-1.42)	0.746	1.02 (0.73-1.42)	0.907	1.06 (0.73-1.54)	0.757
Night shift-workers	1.19 (0.84-1.70)	0.321	1.02 (0.70-1.49)	0.912	0.92 (0.61-1.39)	0.682	1.01 (0.62-1.63)	0.983
Permanent night workers	1.58 (0.86-2.92)	0.144	1.78 (0.93-3.41)	0.081	1.60 (0.77-3.31)	0.204	1.90 (0.79-4.58)	0.155
**High fasting glucose**	*n* = 2301		*n* = 2274		*n = 2066*		*n* = 1731	
Permanent day workers	Ref	-	Ref	-	Ref	-	Ref	-
Day shift-workers	0.89 (0.64-1.25)	0.504	1.05 (0.74-1.50)	0.776	1.07 (0.73-1.58)	0.735	1.04 (0.66-1.63)	0.883
Night shift-workers	1.64 (1.13-2.37)	**0.009**	1.36 (0.91-2.02)	0.135	1.44 (0.93-2.24)	0.106	1.26 (0.74-2.14)	0.389
Permanent night workers	1.87 (0.99-3.51)	0.052	2.14 (1.07-4.29)	**0.031**	1.70 (0.79-3.64)	0.173	1.31 (0.52-3.29)	0.572
**High triglycerides**								
Men	*n* = 1117		*n* = 1116		*n* = 1038		*n* = 886	
Permanent day workers	Ref	-	Ref	-	Ref	-	Ref	-
Day shift-workers	1.16 (0.71-1.88)	0.562	1.14 (0.70-1.87)	0.593	1.30 (0.77-2.19)	0.324	1.32 (0.73-2.40)	0.360
Night shift-workers	0.76 (0.44-1.30)	0.313	0.74 (0.43-1.28)	0.287	0.86 (0.49-1.52)	0.604	0.97 (0.52-1.84)	0.936
Permanent night workers	4.43 (1.70-11.56)	**0.002**	4.31 (1.64-11.30)	**0.003**	3.50 (1.19-10.26)	**0.023**	3.27 (0.99-10.77)	0.051

For peer review only - http://bmjopen.bmj.com/site/about/guidelines.xhtml

Women	*n* = 1109		*n* = 1105		*n* = 1020		*n* = 837	
Permanent day workers	Ref	-	Ref	-	Ref	-	Ref	-
Day shift-workers	1.34 (0.76-2.37)	0.309	1.34 (0.75-2.38)	0.320	1.19 (0.63-2.24)	0.594	0.92 (0.41-2.03)	0.828
Night shift-workers	2.33 (1.09-4.99)	**0.030**	2.29 (1.06-4.95)	**0.035**	2.65 (1.14-6.15)	**0.023**	2.92 (1.03-8.27)	**0.044**
Permanent night workers	1.37 (0.40-4.68)	0.618	1.36 (0.39-4.73)	0.625	1.09 (0.30-3.97)	0.899	0.53 (0.06-4.32)	0.549
**Low HDL-cholesterol**	*n* = 2226		*n* = 2221		*n = 2058*		*n* = 1723	
Permanent day workers	Ref	-	Ref	-	Ref	-	Ref	-
Day shift-workers	0.79 (0.50-1.29)	0.336	0.75 (0.46-1.23)	0.255	0.62 (0.28-1.40)	0.252	0.74 (0.39-1.39)	0.348
Night shift-workers	0.67 (0.35-1.30)	0.240	0.60 (0.31-1.18)	0.138	0.58 (0.29-1.15)	0.116	0.66 (0.30-1.45)	0.300
Permanent night workers	2.22 (1.05-4.71)	**0.038**	1.90 (0.89-4.08)	0.099	1.61 (0.71-3.64)	0.252	1.47 (0.52-4.18)	0.468
**Visceral obesity**								
Men	*n* = 1119		*n* = 1118		*n* = 1043		*n* = 890	
Permanent day workers	Ref	-	Ref	-	Ref	-	Ref	-
Day shift-workers	0.74 (0.42-1.29)	0.288	0.75 (0.42-1.34)	0.333	0.84 (0.47-1.51)	0.561	0.72 (0.36-1.42)	0.341
Night shift-workers	1.18 (0.71-1.94)	0.525	1.11 (0.66-1.84)	0.704	1.06 (0.61-1.85)	0.257	0.84 (0.44-1.63)	0.612
Permanent night workers	5.06 (1.94-13.22)	**0.001**	5.27 (1.99-13.98)	**0.001**	4.79 (1.64-14.03)	**0.004**	3.35 (1.04-10.76)	**0.042**
Women	*n* = 1108		*n* = 1104		*n* = 1022		*n* = 839	
Permanent day workers	Ref	-	Ref	-	Ref	-	Ref	-
Day shift-workers	1.47 (1.01-2.14)	**0.043**	1.48 (1.01-2.17)	**0.043**	1.31 (0.87-1.97)	0.194	1.05 (0.65-1.70)	0.852

For peer review only - http://bmjopen.bmj.com/site/about/guidelines.xhtml

Night shift-workers	1.70 (0.93-3.06)	0.086	1.79 (0.98-3.29)	0.059	1.91 (1.01-3.62)	**0.047**	1.51 (0.66–3.10)	0.324
Permanent night workers	1.70 (0.75-3.84)	0.203	1.69 (0.73-3.92)	0.219	1.75 (0.72-4.23)	0.217	0.83 (0.23-2.99)	0.971

p-values <0.05 are in bold.

Model 1 was adjusted for age (continuous), age square (continuous), sex (except for sex subanalysis) and educational level (middle, low, high). Model 2 was additionally adjusted for weekly alcohol consumption (continuous), smoking status (never, former, current) and for BMI (normal weight, overweight, obese) (except for visceral obesity). Model 3 was additionally adjusted for daily total energy expenditure (continuous).

For peer review only

FIGURE LEGENDS

Figure 1. Multivariable-adjusted risk of metabolic syndrome according to work schedule and sex.

Data are presented on a logarithmic scale and were analyzed using multivariable logistic regression with adjustment for age, educational level, weekly alcohol consumption, smoking status and daily total energy expenditure (Model 3).

For peer review only

For peer review only - http://bmjopen.bmj.com/site/about/guidelines.xhtml

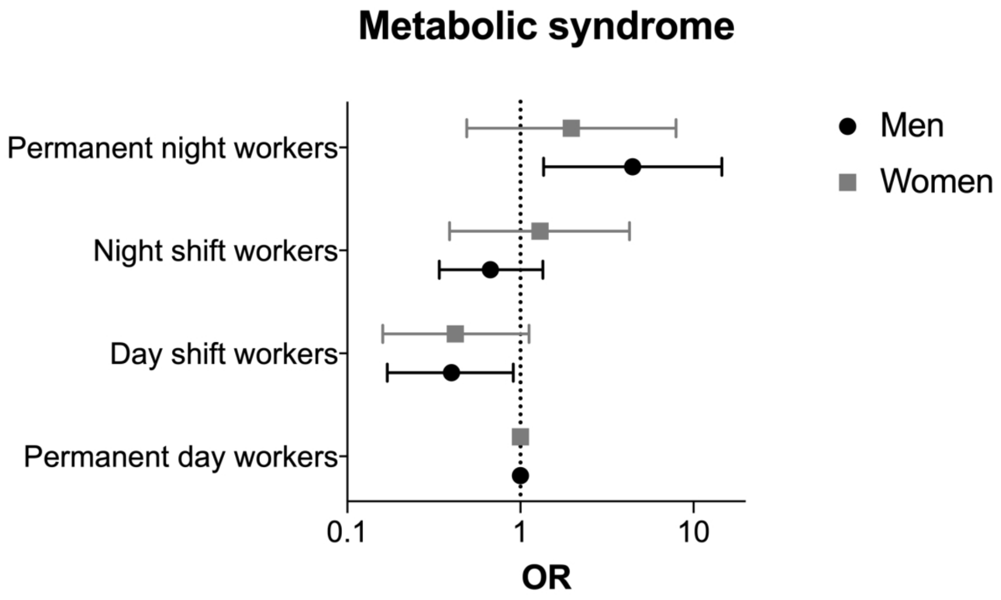

Figure 1. Multivariable-adjusted risk of metabolic syndrome according to work schedule and sex. Data are presented on a logarithmic scale and were analyzed using multivariable logistic regression with adjustment for age, educational level, weekly alcohol consumption, smoking status and daily total energy expenditure (Model 3).

177x109mm (300 x 300 DPI)

SUPPLEMENTAL MATERIAL

Impact of night and shift work on metabolic syndrome and its components in an active middle-aged population-based sample

Virginie Bayon,[1,*] Mathieu Berger,[1,*] Geoffroy Solelhac,[1] José Haba-Rubio,[1] Pedro Marques-Vidal,[2] Marie-Pierre Strippoli,[3] Martin Preisig,[3] Damien Leger,[4,5] Raphael Heinzer[1]

[1]Center for Investigation and Research in Sleep (CIRS), Lausanne University Hospital (CHUV) and University of Lausanne, Lausanne, Switzerland; [2]Department of Medicine, Internal Medicine, Lausanne University Hospital (CHUV) and University of Lausanne, Lausanne, Switzerland; [3]Department of Psychiatry, Lausanne University Hospital (CHUV) and University of Lausanne, Lausanne, Switzerland; [4]Université de Paris, VIFASOM (Equipe d'accueil 5330 VIgilance FAtigue SOMmeil et Santé Publique), Paris, France ; [5]APHP, Hôtel Dieu, Centre du Sommeil et de la Vigilance, Paris, France.

For peer review only

Online Data Supplement:

- **1 Supplementary Figure**
- **1 Supplementary Table**

Supplementary Figure 1. Study flowchart

FU: Follow-up

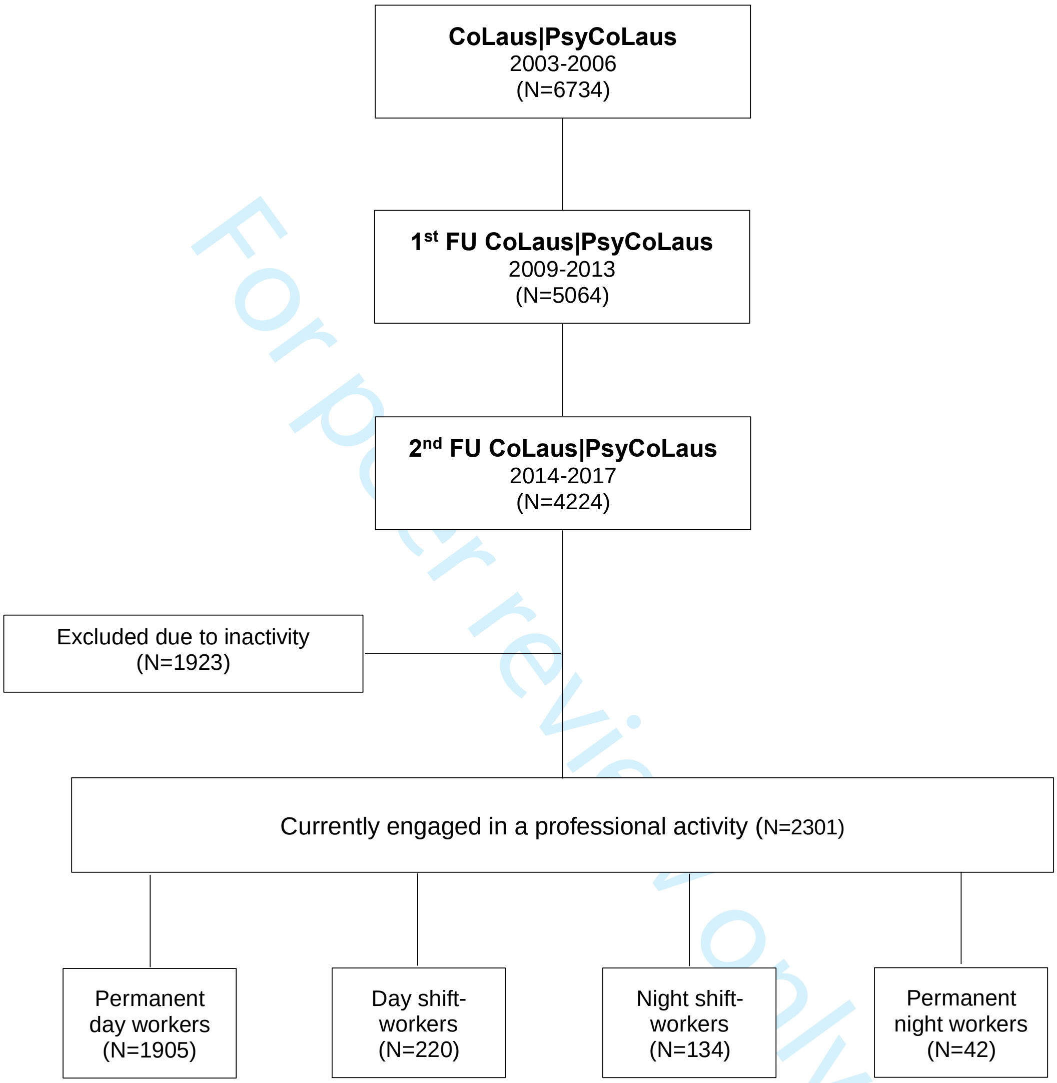

For peer review only

Supplementary Table 1. Association of metabolic syndrome with working schedule

	Crude		Model 1		Model 2	
	N (%)	*p*-value	OR (95% CI)	*p*-value	OR (95% CI)	*p*-value
**Men**	*n* = 1153		*n* = 1059		*n* = 847	
Permanent day workers	Ref	-	Ref	-	Ref	-
Day shift-workers	0.36 (0.18-0.74)	**0.005**	0.33 (0.15-0.69)	**0.004**	0.38 (0.17-0.87)	**0.022**
Night shift-workers	0.78 (0.45-1.34)	0.365	0.71 (0.40-1.26)	0.238	0.67 (0.33-1.36)	0.266
Permanent night workers	6.48 (2.40-17.46)	**<0.001**	6.00 (2.14-16.80)	**0.001**	4.37 (1.33-14.38)	**0.015**
**Women**	*n* = 1148		*n* = 1048		*n* = 798	
Permanent day workers	Ref	-	Ref	-	Ref	-
Day shift-workers	1.17 (0.66-2.05)	0.594	1.15 (0.62-2.12)	0.653	0.47 (0.17-1.24)	0.929
Night shift-workers	1.76 (0.80-3.89)	0.159	1.96 (0.86-4.46)	0.107	1.38 (0.42-4.57)	0.596
Permanent night workers	2.20 (0.81-6.03)	0.124	1.84 (0.60-5.64)	0.289	1.43 (0.28-7.19)	0.668

p-values <0.05 are in bold.

Model 1 was adjusted for age (continuous), age square (continuous) and educational level (middle, low, high). Model 2 was additionally adjusted for weekly alcohol consumption (continuous), smoking status (never, former, current) and daily total energy expenditure.

For peer review only

STROBE Statement—checklist of items that should be included in reports of observational studies

	Item No	Recommendation	Page number
**Title and abstract**	1	(*a*) Indicate the study's design with a commonly used term in the title or the abstract	1, 4
		(*b*) Provide in the abstract an informative and balanced summary of what was done and what was found	4
**Introduction**			
Background/rationale	2	Explain the scientific background and rationale for the investigation being reported	5, 6
Objectives	3	State specific objectives, including any prespecified hypotheses	6
**Methods**			
Study design	4	Present key elements of study design early in the paper	6, 7
Setting	5	Describe the setting, locations, and relevant dates, including periods of recruitment, exposure, follow-up, and data collection	6, 7
Participants	6	(*a*) *Cohort study*—Give the eligibility criteria, and the sources and methods of selection of participants. Describe methods of follow-up<br>*Case-control study*—Give the eligibility criteria, and the sources and methods of case ascertainment and control selection. Give the rationale for the choice of cases and controls<br>*Cross-sectional study*—Give the eligibility criteria, and the sources and methods of selection of participants	6, 7
		(*b*) *Cohort study*—For matched studies, give matching criteria and number of exposed and unexposed<br>*Case-control study*—For matched studies, give matching criteria and the number of controls per case	
Variables	7	Clearly define all outcomes, exposures, predictors, potential confounders, and effect modifiers. Give diagnostic criteria, if applicable	7, 8
Data sources/ measurement	8*	For each variable of interest, give sources of data and details of methods of assessment (measurement). Describe comparability of assessment methods if there is more than one group	*7, 8*
Bias	9	Describe any efforts to address potential sources of bias	9, 10
Study size	10	Explain how the study size was arrived at	7
Quantitative variables	11	Explain how quantitative variables were handled in the analyses. If applicable, describe which groupings were chosen and why	9, 10
Statistical methods	12	(*a*) Describe all statistical methods, including those used to control for confounding	9, 10, 11
		(*b*) Describe any methods used to examine subgroups and interactions	10
		(*c*) Explain how missing data were addressed	10
		(*d*) *Cohort study*—If applicable, explain how loss to follow-up was addressed<br>*Case-control study*—If applicable, explain how matching of cases and controls was addressed<br>*Cross-sectional study*—If applicable, describe analytical methods taking account of sampling strategy	
		(*e*) Describe any sensitivity analyses	

Continued on next page

For peer review only

**Results**			
Participants	13*	(a) Report numbers of individuals at each stage of study—eg numbers potentially eligible, examined for eligibility, confirmed eligible, included in the study, completing follow-up, and analysed	Fig. S1 10
		(b) Give reasons for non-participation at each stage	Fig. S1
		(c) Consider use of a flow diagram	Fig. S1
Descriptive data	14*	(a) Give characteristics of study participants (eg demographic, clinical, social) and information on exposures and potential confounders	11
		(b) Indicate number of participants with missing data for each variable of interest	Table 1
		(c) *Cohort study*—Summarise follow-up time (eg, average and total amount)	
Outcome data	15*	*Cohort study*—Report numbers of outcome events or summary measures over time	
		Case-control study—Report numbers in each exposure category, or summary measures of exposure	
		Cross-sectional study—Report numbers of outcome events or summary measures	*11, 12*
Main results	16	(*a*) Give unadjusted estimates and, if applicable, confounder-adjusted estimates and their precision (eg, 95% confidence interval). Make clear which confounders were adjusted for and why they were included	12, Fig. 1, Table S1
		(*b*) Report category boundaries when continuous variables were categorized	
		(*c*) If relevant, consider translating estimates of relative risk into absolute risk for a meaningful time period	
Other analyses	17	Report other analyses done—eg analyses of subgroups and interactions, and sensitivity analyses	12, 13, Table 3
**Discussion**			
Key results	18	Summarise key results with reference to study objectives	13
Limitations	19	Discuss limitations of the study, taking into account sources of potential bias or imprecision. Discuss both direction and magnitude of any potential bias	16
Interpretation	20	Give a cautious overall interpretation of results considering objectives, limitations, multiplicity of analyses, results from similar studies, and other relevant evidence	16, 17
Generalisability	21	Discuss the generalisability (external validity) of the study results	16, 17
**Other information**			
Funding	22	Give the source of funding and the role of the funders for the present study and, if applicable, for the original study on which the present article is based	18

*Give information separately for cases and controls in case-control studies and, if applicable, for exposed and unexposed groups in cohort and cross-sectional studies.

Note: An Explanation and Elaboration article discusses each checklist item and gives methodological background and published examples of transparent reporting. The STROBE checklist is best used in conjunction with this article (freely

For peer review only - http://bmjopen.bmj.com/site/about/guidelines.xhtml

available on the Web sites of PLoS Medicine at http://www.plosmedicine.org/, Annals of Internal Medicine at http://www.annals.org/, and Epidemiology at http://www.epidem.com/). Information on the STROBE Initiative is available at www.strobe-statement.org.

For peer review only

BMJ Open

Impact of night and shift work on metabolic syndrome and its components: a cross-sectional study in an active middle-aged population-based sample

Journal:	*BMJ Open*
Manuscript ID	bmjopen-2021-053591.R1
Article Type:	Original research
Date Submitted by the Author:	10-Nov-2021
Complete List of Authors:	Bayon, Virginie; CHUV) and University of Lausanne<br>Berger, Mathieu; CHUV, Center for Investigation and Research in Sleep<br>Solelhac, Geoffroy; CHUV) and University of Lausanne<br>Haba-Rubio, José; CHUV) and University of Lausanne<br>Marques-Vidal, Pedro; CHUV) and University of Lausanne, Department of Medicine, Internal Medicine<br>Strippoli, Marie-Pierre; CHUV) and University of Lausanne, Department of Psychiatry<br>Preisig, Martin; CHUV) and University of Lausanne, Department of Psychiatry<br>Leger, Damien; Centre du sommeil et de la vigilance, Hôtel Dieu, APHP; Université Paris Descartes, Sorbonne paris Cité, EA 7330 VIFASOM, Sommeil-Vigilance-Fatigue et Santé Publique,<br>Heinzer, Raphael; CHUV) and University of Lausanne
**Primary Subject Heading**:	Epidemiology
Secondary Subject Heading:	Diabetes and endocrinology, Occupational and environmental medicine
Keywords:	General endocrinology < DIABETES & ENDOCRINOLOGY, EPIDEMIOLOGY, Diabetes & endocrinology < INTERNAL MEDICINE

SCHOLARONE™
Manuscripts

I, the Submitting Author has the right to grant and does grant on behalf of all authors of the Work (as defined in the below author licence), an exclusive licence and/or a non-exclusive licence for contributions from authors who are: i) UK Crown employees; ii) where BMJ has agreed a CC-BY licence shall apply, and/or iii) in accordance with the terms applicable for US Federal Government officers or employees acting as part of their official duties; on a worldwide, perpetual, irrevocable, royalty-free basis to BMJ Publishing Group Ltd ("BMJ") its licensees and where the relevant Journal is co-owned by BMJ to the co-owners of the Journal, to publish the Work in this journal and any other BMJ products and to exploit all rights, as set out in our licence.

The Submitting Author accepts and understands that any supply made under these terms is made by BMJ to the Submitting Author unless you are acting as an employee on behalf of your employer or a postgraduate student of an affiliated institution which is paying any applicable article publishing charge ("APC") for Open Access articles. Where the Submitting Author wishes to make the Work available on an Open Access basis (and intends to pay the relevant APC), the terms of reuse of such Open Access shall be governed by a Creative Commons licence – details of these licences and which Creative Commons licence will apply to this Work are set out in our licence referred to above.

Other than as permitted in any relevant BMJ Author's Self Archiving Policies, I confirm this Work has not been accepted for publication elsewhere, is not being considered for publication elsewhere and does not duplicate material already published. I confirm all authors consent to publication of this Work and authorise the granting of this licence.

For peer review only

Impact of night and shift work on metabolic syndrome and its components: A cross-sectional study in an active middle-aged population-based sample

Short title: Impact of night and shift work

Virginie Bayon, MD[1,*] Mathieu Berger,PhD[1,*] Geoffroy Solelhac,MD[1] José Haba-Rubio,MD[1] Pedro Marques-Vidal,MD[2] Marie-Pierre F. Strippoli,MSc[3] Martin Preisig,MD[3] Damien Leger,MD[4,5] Raphael Heinzer,MD[1]

[1]Center for Investigation and Research in Sleep (CIRS), Lausanne University Hospital (CHUV) and University of Lausanne, Lausanne, Switzerland; [2]Department of Medicine, Internal Medicine, Lausanne University Hospital (CHUV) and University of Lausanne, Lausanne, Switzerland; [3]Department of Psychiatry, Lausanne University Hospital (CHUV) and University of Lausanne, Lausanne, Switzerland; [4]Université de Paris, VIFASOM (Equipe d'accueil 5330 VIgilance FAtigue SOMmeil et Santé Publique), Paris, France ; [5]APHP, Hôtel Dieu, Centre du Sommeil et de la Vigilance, Paris, France

[*]these authors contributed equally to this work

Corresponding Author: Mathieu Berger, PhD, Center for Investigation and Research in Sleep (CIRS), Lausanne University Hospital (CHUV), Rue du Bugnon 46, 1011 Lausanne, Switzerland. Tel: +41 21 314 86 45. E-mail: *mathieuberger@outlook.com*

For peer review only - http://bmjopen.bmj.com/site/about/guidelines.xhtml

For peer review only

Total word count (main text only): 3357.

Number of Tables/Figures: 4/1

For peer review only

For peer review only - http://bmjopen.bmj.com/site/about/guidelines.xhtml

ABSTRACT

Objectives To examine the effects of work schedules on metabolic syndrome and its components in active middle-aged workers.

Methods A cross-sectional analysis including active workers from the population-based CoLaus|PsyCoLaus study (Lausanne, Switzerland) was performed. Work schedule was self-reported and defined as follows: permanent day, day shift, night shift, and permanent night work. Associations between work schedule and the risk of metabolic syndrome and its components were analyzed using multivariable-adjusted logistic regressions.

Results A total of 2301 active workers (mean age 56.2 ± 6.9 years, 50.1% women) were included. Of these, 1905 were permanent day workers, 220 were day shift workers, 134 were night shift workers and 42 were permanent night shift workers. There were significant interactions between sex and work schedule for metabolic syndrome, high triglycerides and visceral obesity. Men but not women permanent night workers had a higher prevalence of metabolic syndrome than permanent day workers in multivariable-adjusted analyses (OR 4.45 [95% CI 1.36-14.56]). Analysis of metabolic syndrome subcomponents showed that the association between work schedule and metabolic syndrome in men was mainly driven by visceral obesity (OR 3.35 [95% CI 1.04-10.76]). Conversely, women but not men working in night shift were at increased risk of having high triglycerides compared with permanent day workers (OR 2.92 [95% CI 1.03-8.27]).

Conclusions The risk of metabolic syndrome is higher in men working in permanent night shift compared with permanent day work and this association could be mediated by visceral obesity.

Keywords work schedule, abdominal obesity, risk factors

For peer review only - http://bmjopen.bmj.com/site/about/guidelines.xhtml
For peer review only

STRENGHS AND LIMITATIONS OF THIS STUDY

- This study evaluated the effects of work schedules on metabolic syndrome and its subcomponent in a general population setting with a precise and extensive assessment of cardio-metabolic phenotypes.
- The association between different shift work schedules and metabolic syndrome was assessed after adjustment for multiple cofounders.
- Because the primary aim of the cohort was not to evaluate the impact of shift work, no precise characterization of workstations and work rhythms (hourly amplitude, direction of rotation, duration of rotations, and duration of exposition) was performed.
- A “healthy worker effect” with a selection of “night shift tolerant” workers cannot be ruled out given the older age of our sample.

For peer review only

Introduction

Due to economic constraints, efficiency needs or performance objectives, night and shift work (3x8) has become highly prevalent in modern societies. Approximately 18% of all European workers work shifts, and this rate is as high as 35% in some countries[1]. Non-standard working schedules (e.g. shift work, night work) are no longer limited to health and safety workers, but are spread across all industries and services, from manufacturing, to transport, telecommunications and more.

Night and shift work interfere with the physiological circadian rhythm, desynchronizing the biological clock, which can favor systemic inflammation[2]. Night and shift work are also associated with reduced and disturbed sleep[3]. Hence, both circadian disruption and short or poor sleep could be mediators explaining the relationship between night or shift work and chronic health conditions, including increased risk of cardiovascular and metabolic disorders[4]. Moreover, several laboratory-controlled studies showed that circadian rhythm desynchronization and sleep restriction have detrimental effects on neuroendocrine, inflammatory and immune functions[5].

The health-related impact of atypical work schedules has thus been a topic of interest for some time[6]. Sleep disturbances, decreased vigilance and increased risk of accidents are among the recognized short-term negative effects of night and shift work[7]. Longer-term health effects have also been described, and include increased risk of cardiovascular and metabolic disorders[8 9]. However, the impact of shift work on metabolic syndrome is not yet completely understood.

Metabolic syndrome combines several interrelated metabolic risk factors associated with all-cause mortality[10]. Subjects with metabolic syndrome have a higher risk of cardiovascular disease mortality and morbidity[11]. Metabolic syndrome definition is

For peer review only - http://bmjopen.bmj.com/site/about/guidelines.xhtml

based on five components: high blood pressure (BP), hyperglycemia, high triglycerides, low high-density lipoprotein (HDL) cholesterol and visceral obesity. A higher prevalence of metabolic syndrome and its components among night and shift workers has previously been suggested in some studies[12 13]. However, the specific effect of shift work and permanent night work remains largely unknown. Moreover, a recent systematic review concluded that there was insufficient evidence regarding the association between shift work and metabolic syndrome when confounding variables are taken into account[14].

Thus, using data of active workers from a population-based study, the aim of the present paper was to assess the cross-sectional association between metabolic syndrome and its components according to four types of work schedules (permanent day, day shift, night shift and permanent night shift work).

Methods

Study design cross-sectional analysis of a population-based cohort study.

Population CoLaus|PsyCoLaus is a population-based cohort exploring the biological, genetic, and environmental determinants of cardiovascular risk factors, cardiovascular diseases, and mental disorders in the population of Lausanne, Switzerland. The methodological aspects (participant recruitment and follow-up) have been previously reported[15]. Briefly, a simple, non-stratified, random sample of 6,734 subjects from the Lausanne population aged 35-75 years was recruited between 2003 and 2006. The baseline and three follow-up evaluations included physical and psychiatric exams, blood sampling, and self-completed questionnaires. All data

For peer review only - http://bmjopen.bmj.com/site/about/guidelines.xhtml

analyzed in the present paper were obtained from the second physical follow-up evaluation (*n* = 4881), which took place between 2014 and 2017.

Patient and Public Involvement

No patients or public were involved in this study design, conduct or analysis.

Exposure and eligibility criteria Professional activity and working hours were self-reported using the following questions: “Are you currently engaged in a professional activity?”; “What is your usual work schedule?” (day exclusively, rotation with no night work, rotation with night work, night work only). The number of work hours per week was also recorded. Participants not currently engaged in a professional activity were excluded from the present analysis. No other exclusion criteria were applied.

Outcome assessment Metabolic syndrome was defined according to the Joint Interim Statement [16] as the presence of at least three of the following five conditions: high BP (systolic BP ≥130 mm Hg or diastolic BP ≥85 mm Hg or use of antihypertensive medication); visceral obesity (waist circumference ≥88 cm in women or ≥102 cm in men); high triglycerides (≥1.7 mmol/L, or use of fibrates or nicotinic acid); low HDL-cholesterol levels (<1.30 mmol/L in women or <1.03 mmol/L in men, or use of fibrates or nicotinic acid); and high fasting plasma glucose (≥5.6 mmol/L or use of anti-diabetic medication). Blood pressure was measured three times on the left arm using an Omron® HEM-907 (Matsusaka, Japan) automated oscillometric sphygmomanometer after at least a 10-min rest in the seated position. The mean of the last two measures was used. Venous blood samples were drawn after an overnight fast to measure the levels of glucose, HDL cholesterol, low HDL-cholesterol, and triglycerides. Biological assays were performed at the clinical

For peer review only - http://bmjopen.bmj.com/site/about/guidelines.xhtml

laboratory of the Lausanne university hospital within two hours of blood collection. Index of insulin resistance during fasting was assessed by the homeostatic model assessment of insulin resistance (HOMA-IR), calculated as the fasting insulin level (in milliunits per milliliter) times the fasting glucose level (in milligrams per liter) divided by 405. Waist circumference was measured twice with a non-stretchable tape over the unclothed abdomen at the mid-point between the lowest rib and the iliac crest. Hip circumference was also measured twice at the greater trochanters. For waist and hip, the mean of the two measurements was used and the waist-to-hip ratio (WHR) was calculated.

Covariates The current socio-professional category was self-reported by participants. Sociodemographic (age, sex) and lifestyle (smoking habit, alcohol intake, coffee consumption) data were collected by self-administered questionnaires. Educational level was categorized as *low* (primary), *middle* (apprenticeship or secondary school) or *high* (university). Smoking status was categorized as *never, former* or *current*. Body weight and height were measured with participants standing without shoes in light indoor clothing. Body weight was measured in kilograms to the nearest 0.1 kg using a Seca™ scale (Seca, Hamburg, Germany). Height was measured to the nearest 5 mm using a Seca™ height gauge (Seca, Hamburg, Germany). Body mass index (BMI) was defined as weight (kg)/height^2 (m^2). Obesity was defined as BMI ≥ 30 kg/m^2.

Medication use was coded according to the World Health Organization Anatomical Therapeutic Chemical (ATC) Classification System (http://www.whocc.no/atcddd). Drugs influencing sleep included hypnotics or sedatives (N05C), anxiolytics (N05B) and antipsychotics (N05A). Diabetes was

For peer review only - http://bmjopen.bmj.com/site/about/guidelines.xhtml

defined as fasting plasma glucose levels ≥7.0 mmol/L or use of antidiabetic medication [17]. Hypertension was defined as systolic BP ≥140 mm Hg and/or diastolic BP ≥90 mm Hg, and/or current use of antihypertensive medication.

The presence of a current major depressive disorder was retrospectively assigned according to Diagnostic and Statistical Manual of Mental Disorders, Fourth Edition (DSM-IV) criteria with information collected at the second and third psychiatric follow-up evaluation using the French translation of the semi-structured Diagnostic Interview for Genetic Studies (DIGS). Cardiovascular disease was defined as previous stroke, heart attack, coronary artery bypass grafting or percutaneous coronary intervention.

Subjective sleep characteristics were determined using the Pittsburgh Sleep Quality Index (PSQI)[18], the Epworth Sleepiness Scale (ESS)[19], and the Berlin questionnaire for sleep-disordered breathing (SDB)[20]. Sleep quality was assessed with the PSQI and dichotomized into good/poor sleep quality (score ≤5/>5), and excessive daytime sleepiness (EDS) was defined as an ESS score >10). A Berlin score ≥2 was defined as indicating a high risk of SDB.

Dietary intake was evaluated using a validated Food Frequency Questionnaire (FFQ) querying the consumption of 97 different food items including portion size over the previous 4 weeks. The daily total energy intake was obtained as well as the proportion of macronutrients, alcohol and fibers.

Physical activity was evaluated with the physical activity frequency questionnaire (PAFQ)[21]. The questionnaire lists 70 types of physical activity from various domains (e.g. occupational, housework, leisure time, sports, etc.) and participants indicated the number of days in the past week (0–7) and the duration per day (0–10 h, in 15-minute increments) for each activity. Energy expenditure

For peer review only - http://bmjopen.bmj.com/site/about/guidelines.xhtml

corresponds to the sum of all the energy expenditure over one week divided by 7 to obtain a mean energy expenditure over a 24-hour period. Sedentary status was defined as spending more than 90% of daily energy in activities below moderate and high intensity (defined as requiring at least 4 times the basal metabolic rate [BMR]). The percentage of total energy >4 metabolic equivalents (METS) was also calculated to quantify moderate and high intensity physical activity.

Statistical analysis Data distribution was graphically assessed using a normal Q-Q plot. Data were presented as number of participants (%) for categorical variables, mean ± SD for normal distribution, or median and interquartile range for non-normally distributed continuous variables. Univariate analyses of continuous data were performed using one-way ANOVA or Kruskal Wallis test follow by Bonferroni's post-hoc or Tamhane's T2 as appropriate. Categorical variables were analyzed using Chi-square test or Fisher's exact test as appropriate. The associations between working schedules (permanent day, day shift work, night shift work and permanent night work) and metabolic syndrome (and its subcomponents) were determined using logistic regression analysis. Prior to this, the interaction of sex with the metabolic syndrome and each of its subcomponents was tested. In case of significant interaction, results were presented for both men and women, otherwise results were shown for the whole sample. Each cardiometabolic risk factor was first tested in univariate analysis (crude) then in two models with serial adjustment for potential confounders. Model 1 was adjusted for age (continuous), educational level (low, middle, high) and sex (except in case of significant sex*outcome interaction). Model 2: Model 1 plus weekly alcohol consumption (continuous), smoking status (never, former, current) and BMI (normal weight, overweight, obese; except for visceral

For peer review only - http://bmjopen.bmj.com/site/about/guidelines.xhtml

obesity). Model 3: Model 2 plus daily total energy expenditure (continuous). Box-Tidwell tests were used to check the assumption of linearity for the logit of each covariate. If the assumption was violated, the square of the covariate was used or the covariate was transformed into categorical variable. To assess collinearity between covariates, a linear regression analysis including all covariates was performed, and the variance inflation factor (VIF) was calculated. A VIF ≤5 was considered as absence of multi-collinearity. Results from logistic regression are presented as OR values with 95% CI. Permanent day workers were considered as the reference group.

All statistical analyses were performed using IBM SPSS Statistics version 26.0 for Macintosh (IBM Corp). Significant results were considered for a two-sided test with $p < 0.05$.

Results

Population characteristics A total of 2301 participants were engaged in a professional activity at the second follow-up of the CoLaus|PsyCoLaus study. Among them, 1905 worked exclusively during the daytime (permanent day workers), 220 were rotation workers with no night work (day shift workers), 134 were rotation workers with night work (night shift workers) and 42 worked exclusively during the night (permanent night workers) (Supplementary Figure 1).

Table 1 and 2 shows the baseline characteristics of the sample according to the four different work schedules. The mean age of the participants was 56.2 ± 6.9 years and half of the sample (50.1%) were women. The proportions of men/women differed significantly according to work schedule: women were more likely to work in day shift and permanent night shift roles, while men were more likely to do night shift

For peer review only - http://bmjopen.bmj.com/site/about/guidelines.xhtml

work. Mean BMI and waist circumference were significantly higher in night shift workers and permanent night workers compared with permanent day workers and day shift workers (p<0.001). Permanent night shift workers were more likely to smoke than other groups, whereas night shift workers were less sedentary than their counterparts. Lipid levels and blood glucose analysis, and sleep parameters in the different work schedule groups are also shown in Table 1 and 2.

Prevalence of metabolic syndrome and its components according to work schedules There were significant interactions between sex and work schedule for metabolic syndrome (p=0.009), high triglycerides (p=0.043) and visceral obesity (p=0.047), but not for high BP, high glucose and low HDL-cholesterol.

The prevalence of the metabolic syndrome was almost three times higher in men permanent night workers compared with men permanent day workers; a similar trend was found for the prevalence of visceral obesity and low HDL-cholesterol (Table 3). The prevalence of high glucose level in night shift workers and permanent night workers was nearly double that in permanent day workers (Table 3).

Association between metabolic syndrome and work schedules by patient sex

Compared to men permanent day workers, permanent night workers showed a higher risk of metabolic syndrome in univariate analysis (OR 6.48 [95% CI 2.40-17.46]; Supplementary Table 1). This significant association persisted after adjustment for age, educational level, alcohol consumption, smoking status and daily total energy expenditure (OR 4.45 [95% CI 1.36-14.56]) (Figure 1). Conversely, the risk of metabolic syndrome in day shift-workers was lower than that in permanent day workers in crude analysis (OR 0.36 [95% CI 0.18-0.74]), and after adjustment in

For peer review only - http://bmjopen.bmj.com/site/about/guidelines.xhtml

models 1 and 2 and 3 (Supplementary Table 1). No significant association between work schedule and metabolic syndrome was found for women.

Association of each component of metabolic syndrome with work schedule

In men, the risk of visceral obesity in permanent night workers was significantly higher than that in permanent day workers, including after adjustment for covariates (Table 4). Moreover, the risk of elevated triglyceride levels in permanent night workers was increased in the crude analysis and after adjustment for age, educational level, alcohol consumption, smoking status and BMI (model 2), but was no longer significant in the fully adjusted model 3 (Table 4).

In women, night shift-workers showed a higher risk of elevated triglyceride levels, which persisted after multiple adjustments (Table 4).

Discussion

In our middle-aged active general population sample, we found differential associations between permanent night work and the risk of metabolic syndrome for men and women. Indeed, permanent night work was only associated with a higher risk of metabolic syndrome in men but not in women. This association could be mediated by a higher risk of visceral obesity in men. The increased risk of metabolic syndrome is in line with previous studies[22]. Some studies even showed that the risk for the development of metabolic syndrome and each of its components gradually and independently increases with accumulated years of shift work[23]. Contrary to other studies, we found no association between permanent night work or night shift-work and metabolic syndrome in women[24 25]. In contrast to the findings on the metabolic syndrome as a whole, for the triglycerides component we found an

For peer review only

increased risk of elevated concentrations among shift workers in women but not in men. This supports previous evidence from Karlsson *et al.* who also reported an elevated triglyceride level among shift workers in 60-year-old women[26].

While the mechanisms underlying the observed increased risk of metabolic syndrome in shift or night workers have not been fully elucidated, several explanatory hypotheses can be proposed. Firstly, sleep duration has been suggested to play a key role in the development of metabolic syndrome. A previous meta-analysis found that short sleep duration was significantly associated with a 27% increase in risk of metabolic syndrome whereas long sleep duration was not[27]. Similar results were found in both men and women. In our study, self-reported sleep duration did not differ between the different groups of workers and therefore does not explain the increased risk of metabolic syndrome observed in permanent night workers among men. However, we cannot rule out that our findings might have been different if objective sleep duration measures were used because objective and subjective sleep duration can differ significantly. Unfortunately, objective sleep assessment could not be included in our analysis. Moreover, sleep fragmentation or an alteration of sleep structure due to irregular sleep schedule or circadian rhythm misalignment in night workers cannot be excluded and could be a possible explanation for the increased risk of metabolic syndrome[28 29].

Secondly, dietary habits could contribute to development of the metabolic syndrome in night or shift workers, but available studies on this subject are scarce. A cross-sectional study comparing 98 rotating shift workers to 100 regular day workers demonstrated that total energy intake and contributions of macronutrients did not differ between the two groups, except for saturated lipids (+10% in shift workers)[30]. However, meal distribution was different in the two groups. Similar to other studies[31]

For peer review only - http://bmjopen.bmj.com/site/about/guidelines.xhtml

[32], we failed to demonstrate a difference in food intake and macronutrients components between night shift workers or permanent night workers compared with permanent day workers. Available data from our study mean that, unfortunately, we cannot rule out the possibility that night shift workers may have had a different circadian distribution of food intake rather than an increase in total daily intake[33].

Thirdly, circadian rhythm desynchronization could be a major contributor to the increased risk of metabolic syndrome among night and shift workers. Still, the underlying pathophysiological mechanisms of this association remain poorly understood. Some animal studies suggested that reduced melatonin production, due to circadian rhythm disruption, could be associated with a higher rate of metabolic syndrome[34]. Furthermore, Fonken *et al.* hypothesized that exposure to light at night altered circadian organization and affected metabolic parameters in mice[35]. Their results emphasized that even weak night lighting (5 lux) is sufficient to desynchronize food consumption and physical activity rhythms, which could explain the observed metabolic disorders[34]. In humans, Corbalan-Tutau *et al.* reported a reduced daily amplitude in melatonin and cortisol circadian patterns associated with metabolic disturbances in women[36]. Unfortunately, we did not measure melatonin and cortisol to confirm these findings in our sample.

With regard to physical activity, we surprisingly found that night shift workers were more active than day shift workers and permanent day workers. This may be due to greater opportunities to perform a physical activity compared with other diurnal workers or to more physically active work among night shift workers, although this should be interpreted with caution due to limited agreement between estimates of activity obtained by PAFQ and those obtained from accelerometers[37].

For peer review only - http://bmjopen.bmj.com/site/about/guidelines.xhtml
For peer review only

Finally, the higher risk of metabolic syndrome we observed in night shift workers may be explained by a vitamin D deficiency[38]. It has been shown that high levels of vitamin D among middle-aged and elderly populations are associated with a substantial decrease in cardiovascular disease, type 2 diabetes and metabolic syndrome[39]. Although we did not measure the vitamin D levels in our different groups of workers, we can hypothesize that permanent night workers have lower exposure to sunlight and may therefore be at higher risk of vitamin D deficiency.

In our study, among the components of the metabolic syndrome, an elevated risk of visceral obesity was found in men permanent night workers. This finding is consistent with a recent meta-analysis which found that shift workers had a higher frequency of abdominal obesity than other obesity types and permanent night workers demonstrated a 29% higher risk of central obesity than rotating shift workers[40].

The main strength of the present study is its large population-based sample with a precise and extensive assessment of cardio-metabolic phenotypes. Indeed, previous studies were mainly performed in specific populations of workers or in particular sectors of activity, such as public health and emergency, which limit the generalizability to other types of shift or night work. In addition, most studies have assessed the risk of metabolic syndrome in shift workers compared with day workers, but few studies have differentiated between shift workers, permanent night workers and shift workers with and without night work.

There are also some limitations that need to be mentioned. First, this study had a cross-sectional design which did not allow to assess causality but only cross-sectional associations that remain to be confirmed in prospective studies. Because the primary aim of the CoLaus|PsyCoLaus study was not to evaluate the impact of

For peer review only - http://bmjopen.bmj.com/site/about/guidelines.xhtml

shift work, the questions related to shift work were only asked at the follow-up 2 (2014-2017), preventing us to investigate longitudinal associations. Moreover, no precise characterization of workstations and work rhythms (hourly amplitude, direction of rotation, duration of rotations, and duration of exposition) was performed. Second, a "healthy worker effect" with a selection of "night shift tolerant" workers cannot be ruled out given the older age of our sample. Third, there were some missing data on self-reported sleep habits and diet parameters and, despite the use of validated questionnaires, declaration bias remains possible. Similarly, only self-reported physical activity was assessed in this study and it would have been interesting to have objective measures of physical activity and sleep to more accurately investigate their influence.

CONCLUSION

Only men permanent night workers were at increased risk of metabolic syndrome compared with permanent day workers, and this association persisted after adjustment for sociodemographic confounders and daily total energy expenditure. From a clinical point of view, we advise monitor of not only BMI but also visceral obesity, particularly in men permanent night workers. Further prospective studies are needed to confirm theses cross-sectional results and elucidate the underline mechanisms.

For peer review only - http://bmjopen.bmj.com/site/about/guidelines.xhtml

ARTICLE INFORMATION

Acknowledgments

The authors thank Prof. Mehdi Tafti, Prof. Vincent Mooser, Daniela Andries and Nadia Tobback for their important contribution to the HypnoLaus and CoLaus|PsyCoLaus Cohorts, the Lausanne population who volunteered to participate in the CoLaus|PsyCoLaus and HypnoLaus studies, as well as the whole team of CoLaus|PsyCoLaus.

For peer review only

Funding

The HypnoLaus and the CoLaus/ PsyCoLaus studies were and are supported by research grants from GlaxoSmithKline ('not applicable'), the Faculty of Biology and Medicine of Lausanne ('not applicable'), the Swiss National Science Foundation (grants 3200B0-105993, 3200B0-118308, 33CSCO-122661, 33CS30-139468 and 33CS30- 148401), Leenaards Foundation ('not applicable'), and Vaud Pulmonary League ('not applicable'). The contents of this research are solely the responsibility of the authors.

Author Disclosures

The authors have no conflicts of interest to declare.

Contributors

VB, MB, PMV, MP and RH designed the study. JHR, PMV, MPFS, MP and RH collected the data. MB performed the statistical analysis. VB, MB, GS, JHR, PMV, MPFS, MP, DL and RH interpreted the data. VB and MB wrote the first draft of the manuscript and GS, JHR, PMV, MPFS, MP, DL and RH critically reviewed the

For peer review only - http://bmjopen.bmj.com/site/about/guidelines.xhtml

manuscript. All authors undertake to give final approval of the version to be published and agree to be accountable for all aspects of the work. VB is the guarantor of this work and, as such, had full access to all the data in the study and takes responsibility for the integrity of the data and the accuracy of the data analysis.

Data availability statement

Data may be obtained from a third party and are not publicly available. All data relevant to the study are included in the article or uploaded as supplementary information.

Ethics statements

1. Patient consent for publication

Not applicable.

2. Ethics approval

The study was approved by the Institutional Ethics Committee of the University of Lausanne (decision reference 33/09) and written inform consent was obtained from all subjects. A copy of the written Inform Consent form was handed out to the subjects. A further copy was provided for the archives of the study in the Center for Investigation and Research in Sleep (CIRS, Lausanne University Hospital, Switzerland).

For peer review only

References

1 Nappo N. Is there an association between working conditions and health? An analysis of the Sixth European Working Conditions Survey data. *PLoS One* 2019;14:e0211294.

2 Puttonen S, Viitasalo K, Harma M. Effect of shiftwork on systemic markers of inflammation. *Chronobiol Int* 2011;28:528-35.

3 Sallinen M, Kecklund G. Shift work, sleep, and sleepiness - differences between shift schedules and systems. *Scand J Work Environ Health* 2010;36:121-33.

4 Knutson KL, Spiegel K, Penev P, Van Cauter E. The metabolic consequences of sleep deprivation. *Sleep Med Rev* 2007;11:163-78.

5 Faraut B, Bayon V, Leger D. Neuroendocrine, immune and oxidative stress in shift workers. *Sleep Med Rev* 2013;17:433-44.

6 Kecklund G, Axelsson J. Health consequences of shift work and insufficient sleep. *BMJ* 2016;355:i5210.

7 Drake CL, Roehrs T, Richardson G, Walsh JK, Roth T. Shift work sleep disorder: prevalence and consequences beyond that of symptomatic day workers. *Sleep* 2004;27:1453-62.

8 Morikawa Y, Nakagawa H, Miura K et al. Effect of shift work on body mass index and metabolic parameters. *Scand J Work Environ Health* 2007;33:45-50.

9 Wang D, Ruan W, Chen Z, Peng Y, Li W. Shift work and risk of cardiovascular disease morbidity and mortality: A dose-response meta-analysis of cohort studies. *Eur J Prev Cardiol* 2018;25:1293-1302.

10 Wu SH, Liu Z, Ho SC. Metabolic syndrome and all-cause mortality: a meta-analysis of prospective cohort studies. *Eur J Epidemiol* 2010;25:375-84.

For peer review only - http://bmjopen.bmj.com/site/about/guidelines.xhtml

11 Galassi A, Reynolds K, He J. Metabolic syndrome and risk of cardiovascular disease: a meta-analysis. *Am J Med* 2006;119:812-9.

12 Karlsson B, Knutsson A, Lindahl B. Is there an association between shift work and having a metabolic syndrome? Results from a population based study of 27,485 people. *Occup Environ Med* 2001;58:747-52.

13 Violanti JM, Burchfiel CM, Hartley TA et al. Atypical work hours and metabolic syndrome among police officers. *Arch Environ Occup Health* 2009;64:194-201.

14 Canuto R, Garcez AS, Olinto MT. Metabolic syndrome and shift work: a systematic review. *Sleep Med Rev* 2013;17:425-31.

15 Firmann M, Mayor V, Vidal PM et al. The CoLaus study: a population-based study to investigate the epidemiology and genetic determinants of cardiovascular risk factors and metabolic syndrome. *BMC Cardiovasc Disord* 2008;8:6.

16 Alberti KG, Eckel RH, Grundy SM et al. Harmonizing the metabolic syndrome: a joint interim statement of the International Diabetes Federation Task Force on Epidemiology and Prevention; National Heart, Lung, and Blood Institute; American Heart Association; World Heart Federation; International Atherosclerosis Society; and International Association for the Study of Obesity. *Circulation* 2009;120:1640-5.

17 American Diabetes A. Diagnosis and classification of diabetes mellitus. *Diabetes Care* 2014;37 Suppl 1:S81-90.

18 Buysse DJ, Reynolds CF, 3rd, Monk TH, Berman SR, Kupfer DJ. The Pittsburgh Sleep Quality Index: a new instrument for psychiatric practice and research. *Psychiatry Res* 1989;28:193-213.

19 Johns MW. A new method for measuring daytime sleepiness: the Epworth sleepiness scale. *Sleep* 1991;14:540-5.

For peer review only - http://bmjopen.bmj.com/site/about/guidelines.xhtml

20 Netzer NC, Stoohs RA, Netzer CM, Clark K, Strohl KP. Using the Berlin Questionnaire to identify patients at risk for the sleep apnea syndrome. *Ann Intern Med* 1999;131:485-91.

21 Bernstein M, Sloutskis D, Kumanyika S, Sparti A, Schutz Y, Morabia A. Data-based approach for developing a physical activity frequency questionnaire. *Am J Epidemiol* 1998;147:147-54.

22 Wang F, Zhang L, Zhang Y et al. Meta-analysis on night shift work and risk of metabolic syndrome. *Obes Rev* 2014;15:709-20.

23 De Bacquer D, Van Risseghem M, Clays E, Kittel F, De Backer G, Braeckman L. Rotating shift work and the metabolic syndrome: a prospective study. *Int J Epidemiol* 2009;38:848-54.

24 Lim YC, Hoe VCW, Darus A, Bhoo-Pathy N. Association between night-shift work, sleep quality and metabolic syndrome. *Occup Environ Med* 2018;75:716-723.

25 Guo Y, Rong Y, Huang X et al. Shift work and the relationship with metabolic syndrome in Chinese aged workers. *PLoS One* 2015;10:e0120632.

26 Karlsson B, Knutsson A, Lindahl B. Is there an association between shift work and having a metabolic syndrome? Results from a population based study of 27,485 people. *Occupational and Environmental Medicine* 2001;58:747-752.

27 Xi B, He D, Zhang M, Xue J, Zhou D. Short sleep duration predicts risk of metabolic syndrome: a systematic review and meta-analysis. *Sleep Med Rev* 2014;18:293-7.

28 Stamatakis KA, Punjabi NM. Effects of sleep fragmentation on glucose metabolism in normal subjects. *Chest* 2010;137:95-101.

29 Tasali E, Leproult R, Ehrmann DA, Van Cauter E. Slow-wave sleep and the risk of type 2 diabetes in humans. *Proc Natl Acad Sci U S A* 2008;105:1044-9.

For peer review only

For peer review only - http://bmjopen.bmj.com/site/about/guidelines.xhtml

30 Esquirol Y, Bongard V, Mabile L, Jonnier B, Soulat JM, Perret B. Shift work and metabolic syndrome: respective impacts of job strain, physical activity, and dietary rhythms. *Chronobiol Int* 2009;26:544-59.

31 de Assis MA, Kupek E, Nahas MV, Bellisle F. Food intake and circadian rhythms in shift workers with a high workload. *Appetite* 2003;40:175-83.

32 Morikawa Y, Miura K, Sasaki S et al. Evaluation of the effects of shift work on nutrient intake: a cross-sectional study. *J Occup Health* 2008;50:270-8.

33 Molzof HE, Wirth MD, Burch JB et al. The impact of meal timing on cardiometabolic syndrome indicators in shift workers. *Chronobiol Int* 2017;34:337-348.

34 Fonken LK, Workman JL, Walton JC et al. Light at night increases body mass by shifting the time of food intake. *Proc Natl Acad Sci U S A* 2010;107:18664-9.

35 Fonken LK, Nelson RJ. The effects of light at night on circadian clocks and metabolism. *Endocr Rev* 2014;35:648-70.

36 Corbalan-Tutau D, Madrid JA, Nicolas F, Garaulet M. Daily profile in two circadian markers "melatonin and cortisol" and associations with metabolic syndrome components. *Physiol Behav* 2014;123:231-5.

37 Verhoog S, Gubelmann C, Guessous I, Bano A, Franco OH, Marques-Vidal P. Comparison of the Physical Activity Frequency Questionnaire (PAFQ) with accelerometry in a middle-aged and elderly population: The CoLaus study. *Maturitas* 2019;129:68-75.

38 Ju SY, Jeong HS, Kim DH. Blood vitamin D status and metabolic syndrome in the general adult population: a dose-response meta-analysis. *J Clin Endocrinol Metab* 2014;99:1053-63.

39 Parker J, Hashmi O, Dutton D et al. Levels of vitamin D and cardiometabolic disorders: systematic review and meta-analysis. *Maturitas* 2010;65:225-36.

For peer review only - http://bmjopen.bmj.com/site/about/guidelines.xhtml

40 Sun M, Feng W, Wang F et al. Meta-analysis on shift work and risks of specific obesity types. *Obes Rev* 2018;19:28-40.

For peer review only

For peer review only - http://bmjopen.bmj.com/site/about/guidelines.xhtml

Table 1. Baseline characteristics according to work schedules

	Permanent day workers (*n* = 1905)	Day shift workers (*n* = 220)	Night shift workers (*n* = 134)	Permanent night workers (*n* = 42)	*p*-value	N Total
**Demographics & anthropometrics**						
Age (years)	55.0 (50.0–60.0)	55.0 (50.5–59.5)	54.5 (50.4–58.6)	53.0 (48.8–57.2)	0.070	2275
Men, n (%)	958 (50.3)	89 (40.5) [a]	88 (65.7) [a]	18 (42.9)	**<0.001**	2301
Educational level, n (%)					**<0.001**	2300
Low	791 (41.5)	112 (50.9)	71 (53.0)	30 (71.4) [a]		
High	591 (31.0) [a]	43 (19.5)	23 (17.2)	2 (4.8)		
Body-mass index (kg/m^2)	25.4 (22.6–28.5)	25.5 (23.1–27.6)	26.0 (23.2–30.0) [b]	27.9 (25.4–31.3) [b,c]	**<0.001**	2228
Waist circumference (cm)	89.5 (81.0–98.5)	89.0 (81.4–96.0)	93.0 (84.3–102.0) [b,c]	95.0 (85.3–109.0) [b,c]	**<0.001**	2227
Waist to hip ratio	0.88 ± 0.09	0.87 ± 0.08	0.90 ± 0.09 [b,c]	0.90 ± 0.09	**0.013**	2227
**Risk factors**						
Metabolic syndrome, n (%)	327 (17.2)	25 (11.4)	25 (18.7)	17 (40.5)[a]	**<0.001**	2301
Number of metabolic risk factors†	1 (0–2)	1 (0–2)	1 (0–2)	2 (1–3) [b,c]	**0.006**	2301
Current major depressive disorder, n (%)	115 (7.9)	19 (11.7)	10 (9.6)	4 (12.1)	0.319	1756
Hypertension, n (%)	653 (34.9)	80 (36.7)	45 (33.6)	12 (28.6)	0.764	2263
Diabetes, n (%)	100 (5.4)	8 (3.7)	18 (13.6) [a]	4 (9.5)	**<0.001**	2231
Dyslipidemia, n (%)	413 (22.5)	51 (23.6)	29 (22.1)	16 (38.1)	0.123	2226
Sleep drugs, n (%)	109 (5.7)	14 (6.4)	4 (3.0)	4 (9.5)	0.367	2301
Cardiovascular disease, n (%)††	55 (2.9)	12 (5.5)	5 (3.7)	3 (7.1)	0.102	2291
**Risk factors**						
Smoking status, n (%)					**0.011**	2246
Former	689 (37.0)	86 (40.8)	57 (43.8)	9 (22.0) [a]		
Curent	404 (21.7)	44 (20.9)	22 (16.9)	18 (43.9) [a]		

For peer review only - http://bmjopen.bmj.com/site/about/guidelines.xhtml
For peer review only

Alcohol (units/week)	4 (1–9)	3 (0–7)	3 (0–7)	2 (0–6)	**0.010**	2162
Coffee consumption, n (%)					0.961	2222
None	186 (10.1)	23 (11.0)	13 (10.2)	4 (9.8)		
1-3 cups/day	1154 (62.6)	134 (64.1)	78 (60.9)	28 (68.3)		
≥4 cups/day	504 (27.3)	52 (24.9)	37 (28.9)	9 (22.0)		
Total energy intake (Kcals/day)	1756 ± 664	1761 ± 654	1828 ± 719	1853 ± 619	0.603	1996
Physical activity						
Total energy expenditure (Kcals/day)	2656 (2297–3076)	2698 (2336–3046)	3118 (2735–3578) [b,c]	2663 (2356–3164)	**<0.001**	1828
Activity ≥4 MET (% total activity)	10.1 (1.9–18.4)	8.8 (1.2–20.2)	14.4 (4.9–25.3) [b]	6.5 (0.3–16.1)	**0.005**	1828
Sedentary status, n (%)	758 (49.2)	92 (55.1)	32 (34.4) [a]	16 (57.1)	**0.011**	1828
**Blood analysis**						
Total cholesterol (mmol/L)	5.3 ± 0.9	5.4 ± 0.9	5.3 ± 0.9	5.4 ± 1.0	0.928	2226
HDL cholesterol (mmol/L)	1.5 (1.2–1.9)	1.6 (1.3–1.9)	1.4 (1.2–1.8)	1.4 (1.1–1.7)	**0.013**	2226
LDL cholesterol (mmol/L)	3.2 ± 0.8	3.2 ± 0.8	3.2 ± 0.8	3.2 ± 0.9	0.958	2226
Triglycerides (mmol/L)	1.0 (0.8–1.4)	1.0 (0.8–1.5)	1.1 (0.8–1.5)	1.2 (0.9–1.8)	0.278	2226
Fasting glucose (mmol/L)	5.2 (4.9–5.5)	5.1 (4.8–5.5)	5.2 (4.9–5.8)	5.5 (5–5.9)	**0.026**	2226
Insulin (microIU/mL)	7 (4.8–10.6)	7.2 (4.6–10.9)	7.3 (5.0–11.7)	8.8 (6.5–12.9)	**0.027**	2218
HOMA-IR[†††]	1.6 (1.1–2.6)	1.6 (1.0–2.6)	1.7 (1.2–3.2)	2.1 (1.5–3.4)	**0.012**	2218

Data are presented as mean ± SD or median and interquartile range for continuous variables and number of participants (%) for categorical variables. P-value < 0.05 are shown in bold. [a] adjusted residual >│ 2│; [b] statistically different from "day only"; [c] statistically different from "shift work without night". † Metabolic risk factor corresponded to the five risk factors which defined the metabolic syndrome according to the Joint Interim Statement [16]: Systolic blood pressure ≥130 mmHg or diastolic blood pressure ≥85 mmHg or use of antihypertensive medication; waist circumference ≥88 cm in women or ≥102 cm in men); triglycerides ≥1.7 mmol/L, or use of fibrates or nicotinic acid; HDL-cholesterol <1.30 mmol/L in women or <1.03 mmol/L in men, or use of fibrates or nicotinic acid; and high fasting plasma glucose (≥5.6 mmol/L or use of anti-diabetic

For peer review only - http://bmjopen.bmj.com/site/about/guidelines.xhtml
For peer review only

medication). †† Cardiovascular disease was defined by previous stroke, heart attack, coronary artery bypass grafting or percutaneous coronary intervention. ††† Index of insulin resistance during fasting was assessed by the homeostatic model assessment of insulin resistance (HOMA-IR), calculated as the fasting insulin level (in milliunits per milliliter) times the fasting glucose level (in milligrams per liter) divided by 405.

HDL: high density lipoprotein; LDL: low density lipoprotein; MET: metabolic equivalent of task.

Table 2. Working and sleep characteristics according to work schedules

	**Permanent day workers (*n* = 1905)**	**Day shift workers (*n* = 220)**	**Night shift workers (*n* = 134)**	**Permanent night workers (*n* = 42)**	***p*-value**	**N Total**
**Working characteristics**						
Number of working hours/week	38.0 ± 14.7	38.7 ± 15.2	43.1 ± 18.1	38.0 ± 15.2	0.260	2285
Work time, n (%)					0.397	2258
Full-time	1569 (83.8)	181 (84.6)	111 (86.0)	39 (92.9)		
<50%	304 (16.2)	33 (15.4)	18 (14.0)	3 (7.1)		
Example of physical intensity at work, n (%)					**<0.001**	2135
Sedentary (sitting/driving)	1409 (79.5)	105 (51.2)	66 (55.0)	14 (37.8)		
Pushing wheelbarrow	283 (16.0)	81 (39.5)	40 (33.3)	16 (43.2)		
Unloading a truck without assist.	81 (4.6)	19 (9.3)	14 (11.7)	7 (18.9)		
**Sleep & vigilance**						
Epworth Sleepiness Scale score	6 (4–8)	5 (3–8)	6 (4–9)	5 (3–8)	0.623	1786
Excessive daytime sleepiness, n (%)‡	182 (12.1)	20 (12.6)	14 (14.0)	3 (11.1)	0.950	1786
Poor sleep quality, n (%)‡‡	415 (31.5)	46 (37.4)	27 (32.1)	7 (35.0)	0.600	1542
High risk of SDB, n (%)§	321 (21.3)	34 (21.0)	29 (28.4)	8 (27.6)	0.323	1800
Self-reported total sleep time (h)	6.9 ± 1.0	6.8 ± 0.9	6.9 ± 1.0	7.1 ± 1.3	0.507	1542

For peer review only - http://bmjopen.bmj.com/site/about/guidelines.xhtml

Data are presented as mean ± SD or median and interquartile range for continuous variables and number of participants (%) for categorical variables. P-value < 0.05 are shown in bold. [‡] Excessive daytime sleepiness was defined by an Epworth Sleepiness Scale score >10; [‡‡] Poor sleep quality was defined by a Pittsburgh Sleep Quality Index score >5; [§] High risk of SDB was defined by a Berlin score >2.

For peer review only

For peer review only - http://bmjopen.bmj.com/site/about/guidelines.xhtml

Table 3. Prevalence of metabolic syndrome and its subcomponents according to work schedule

	**Permanent day workers (*n* = 1905)**	**Day shift workers (*n* = 220)**	**Night shift workers (*n* = 134)**	**Permanent night workers (*n* = 42)**	***p*-value**
Metabolic syndrome					
Men	226 (23.6)	9 (10.1)	17 (19.3)	12 (66.7)	**<0.001**
Women	101 (10.7)	16 (12.2)	8 (17.4)	5 (20.8)	0.225
High BP	826 (43.4)	91 (41.4)	64 (47.8)	23 (54.8)	0.313
High glucose	472 (24.8)	50 (22.7)	47 (35.1)	16 (38.1)	**0.010**
High triglycerides					
Men	243 (26.2)	25 (29.1)	18 (21.2)	11 (61.1)	**0.006**
Women	86 (9.5)	16 (12.3)	9 (19.6)	3 (12.5)	0.183
Low HDL-cholesterol	201 (10.9)	19 (8.8)	10 (7.6)	9 (21.4)	0.064
Visceral obesity					
Men	220 (23.7)	16 (18.6)	23 (26.7)	11 (61.1)	**0.002**
Women	302 (33.3)	55 (42.3)	21 (45.7)	11 (45.8)	0.051

Data are presented as n (%).

Where there was an interaction of outcome*sex, results are presented separately for men and women, otherwise for the whole cohort.

For peer review only

For peer review only - http://bmjopen.bmj.com/site/about/guidelines.xhtml

Table 4. Association of each component of the metabolic syndrome with work schedule

	Crude		Model 1		Model 2		Model 3	
	**OR (95% CI)**	***p*-value**	**OR (95% CI)**	***p*-value**	**OR (95% CI)**	***p*-value**	**OR (95% CI)**	***p*-value**
**High BP**	*n* = 2301		*n* = 2274		*n = 2066*		*n* = 1731	
Day shift-workers	0.92 (0.69-1.22)	0.572	1.05 (0.78-1.42)	0.746	1.02 (0.73-1.42)	0.907	1.06 (0.73-1.54)	0.757
Night shift-workers	1.19 (0.84-1.70)	0.321	1.02 (0.70-1.49)	0.912	0.92 (0.61-1.39)	0.682	1.01 (0.62-1.63)	0.983
Permanent night workers	1.58 (0.86-2.92)	0.144	1.78 (0.93-3.41)	0.081	1.60 (0.77-3.31)	0.204	1.90 (0.79-4.58)	0.155
**High fasting glucose**	*n* = 2301		*n* = 2274		*n = 2066*		*n* = 1731	
Day shift-workers	0.89 (0.64-1.25)	0.504	1.05 (0.74-1.50)	0.776	1.07 (0.73-1.58)	0.735	1.04 (0.66-1.63)	0.883
Night shift-workers	1.64 (1.13-2.37)	**0.009**	1.36 (0.91-2.02)	0.135	1.44 (0.93-2.24)	0.106	1.26 (0.74-2.14)	0.389
Permanent night workers	1.87 (0.99-3.51)	0.052	2.14 (1.07-4.29)	**0.031**	1.70 (0.79-3.64)	0.173	1.31 (0.52-3.29)	0.572
**High triglycerides**								
Men	*n* = 1117		*n* = 1116		*n* = 1038		*n* = 886	
Day shift-workers	1.16 (0.71-1.88)	0.562	1.14 (0.70-1.87)	0.593	1.30 (0.77-2.19)	0.324	1.32 (0.73-2.40)	0.360
Night shift-workers	0.76 (0.44-1.30)	0.313	0.74 (0.43-1.28)	0.287	0.86 (0.49-1.52)	0.604	0.97 (0.52-1.84)	0.936
Permanent night workers	4.43 (1.70-11.56)	**0.002**	4.31 (1.64-11.30)	**0.003**	3.50 (1.19-10.26)	**0.023**	3.27 (0.99-10.77)	0.051
Women	*n* = 1109		*n* = 1105		*n* = 1020		*n* = 837	
Day shift-workers	1.34 (0.76-2.37)	0.309	1.34 (0.75-2.38)	0.320	1.19 (0.63-2.24)	0.594	0.92 (0.41-2.03)	0.828
Night shift-workers	2.33 (1.09-4.99)	**0.030**	2.29 (1.06-4.95)	**0.035**	2.65 (1.14-6.15)	**0.023**	2.92 (1.03-8.27)	**0.044**
Permanent night workers	1.37 (0.40-4.68)	0.618	1.36 (0.39-4.73)	0.625	1.09 (0.30-3.97)	0.899	0.53 (0.06-4.32)	0.549
**Low HDL-cholesterol**	*n* = 2226		*n* = 2221		*n = 2058*		*n* = 1723	
Day shift-workers	0.79 (0.50-1.29)	0.336	0.75 (0.46-1.23)	0.255	0.62 (0.28-1.40)	0.252	0.74 (0.39-1.39)	0.348

For peer review only - http://bmjopen.bmj.com/site/about/guidelines.xhtml

Night shift-workers	0.67 (0.35-1.30)	0.240	0.60 (0.31-1.18)	0.138	0.58 (0.29-1.15)	0.116	0.66 (0.30-1.45)	0.300
Permanent night workers	2.22 (1.05-4.71)	**0.038**	1.90 (0.89-4.08)	0.099	1.61 (0.71-3.64)	0.252	1.47 (0.52-4.18)	0.468
**Visceral obesity**								
Men	*n* = 1119		*n* = 1118		*n* = 1043		*n* = 890	
Day shift-workers	0.74 (0.42-1.29)	0.288	0.75 (0.42-1.34)	0.333	0.84 (0.47-1.51)	0.561	0.72 (0.36-1.42)	0.341
Night shift-workers	1.18 (0.71-1.94)	0.525	1.11 (0.66-1.84)	0.704	1.06 (0.61-1.85)	0.257	0.84 (0.44-1.63)	0.612
Permanent night workers	5.06 (1.94-13.22)	**0.001**	5.27 (1.99-13.98)	**0.001**	4.79 (1.64-14.03)	**0.004**	3.35 (1.04-10.76)	**0.042**
Women	*n* = 1108		*n* = 1104		*n* = 1022		*n* = 839	
Day shift-workers	1.47 (1.01-2.14)	**0.043**	1.48 (1.01-2.17)	**0.043**	1.31 (0.87-1.97)	0.194	1.05 (0.65-1.70)	0.852
Night shift-workers	1.70 (0.93-3.06)	0.086	1.79 (0.98-3.29)	0.059	1.91 (1.01-3.62)	**0.047**	1.51 (0.66–3.10)	0.324
Permanent night workers	1.70 (0.75-3.84)	0.203	1.69 (0.73-3.92)	0.219	1.75 (0.72-4.23)	0.217	0.83 (0.23-2.99)	0.971

Data are presented as odds ratio (OR) and 95% confidence intervals (CI). For each component analyzed, the "permanent day workers" were considered as the reference group. *p*-values <0.05 are in bold. Model 1 was adjusted for age (continuous), age square (continuous), sex (except for sex subanalysis) and educational level (middle, low, high). Model 2 was additionally adjusted for weekly alcohol consumption (continuous), smoking status (never, former, current) and for BMI (normal weight, overweight, obese) (except for visceral obesity). Model 3 was additionally adjusted for daily total energy expenditure (continuous).

For peer review only - http://bmjopen.bmj.com/site/about/guidelines.xhtml

FIGURE LEGENDS

Figure 1. Multivariable-adjusted risk of metabolic syndrome according to work schedule and sex.

Data are presented on a logarithmic scale and were analyzed using multivariable logistic regression with adjustment for age, educational level, weekly alcohol consumption, smoking status and daily total energy expenditure (Model 3).

For peer review only

For peer review only - http://bmjopen.bmj.com/site/about/guidelines.xhtml

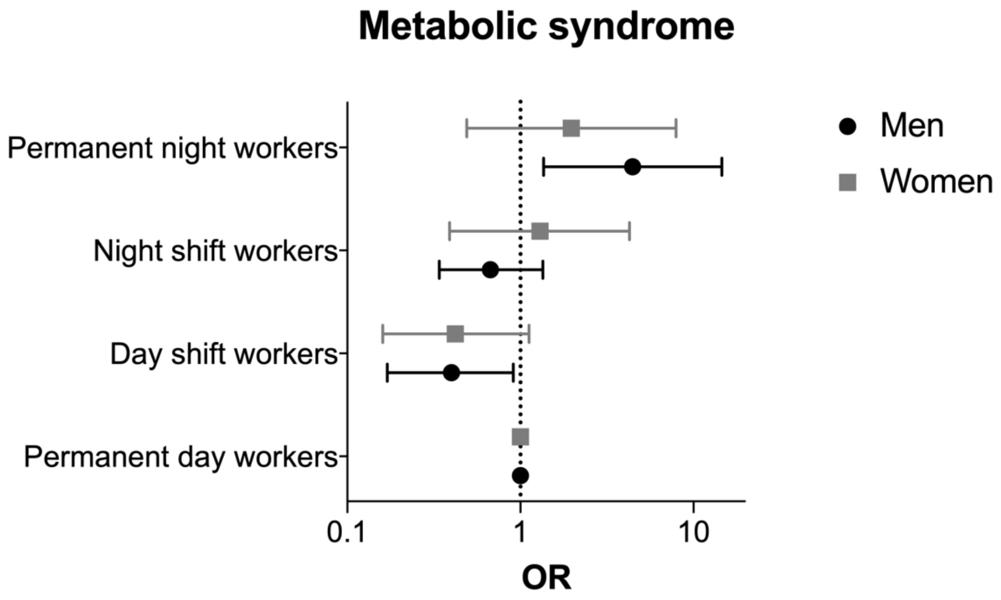

Figure 1. Multivariable-adjusted risk of metabolic syndrome according to work schedule and sex. Data are presented on a logarithmic scale and were analyzed using multivariable logistic regression with adjustment for age, educational level, weekly alcohol consumption, smoking status and daily total energy expenditure (Model 3).

177x109mm (300 x 300 DPI)

SUPPLEMENTAL MATERIAL

Impact of night and shift work on metabolic syndrome and its components in an active middle-aged population-based sample

Virginie Bayon,[1,*] Mathieu Berger,[1,*] Geoffroy Solelhac,[1] José Haba-Rubio,[1] Pedro Marques-Vidal,[2] Marie-Pierre Strippoli,[3] Martin Preisig,[3] Damien Leger,[4,5] Raphael Heinzer[1]

[1]Center for Investigation and Research in Sleep (CIRS), Lausanne University Hospital (CHUV) and University of Lausanne, Lausanne, Switzerland; [2]Department of Medicine, Internal Medicine, Lausanne University Hospital (CHUV) and University of Lausanne, Lausanne, Switzerland; [3]Department of Psychiatry, Lausanne University Hospital (CHUV) and University of Lausanne, Lausanne, Switzerland; [4]Université de Paris, VIFASOM (Equipe d'accueil 5330 VIgilance FAtigue SOMmeil et Santé Publique), Paris, France ; [5]APHP, Hôtel Dieu, Centre du Sommeil et de la Vigilance, Paris, France.

For peer review only

Online Data Supplement:

- **1 Supplementary Figure**
- **1 Supplementary Table**

Supplementary Figure 1. Study flowchart

FU: Follow-up

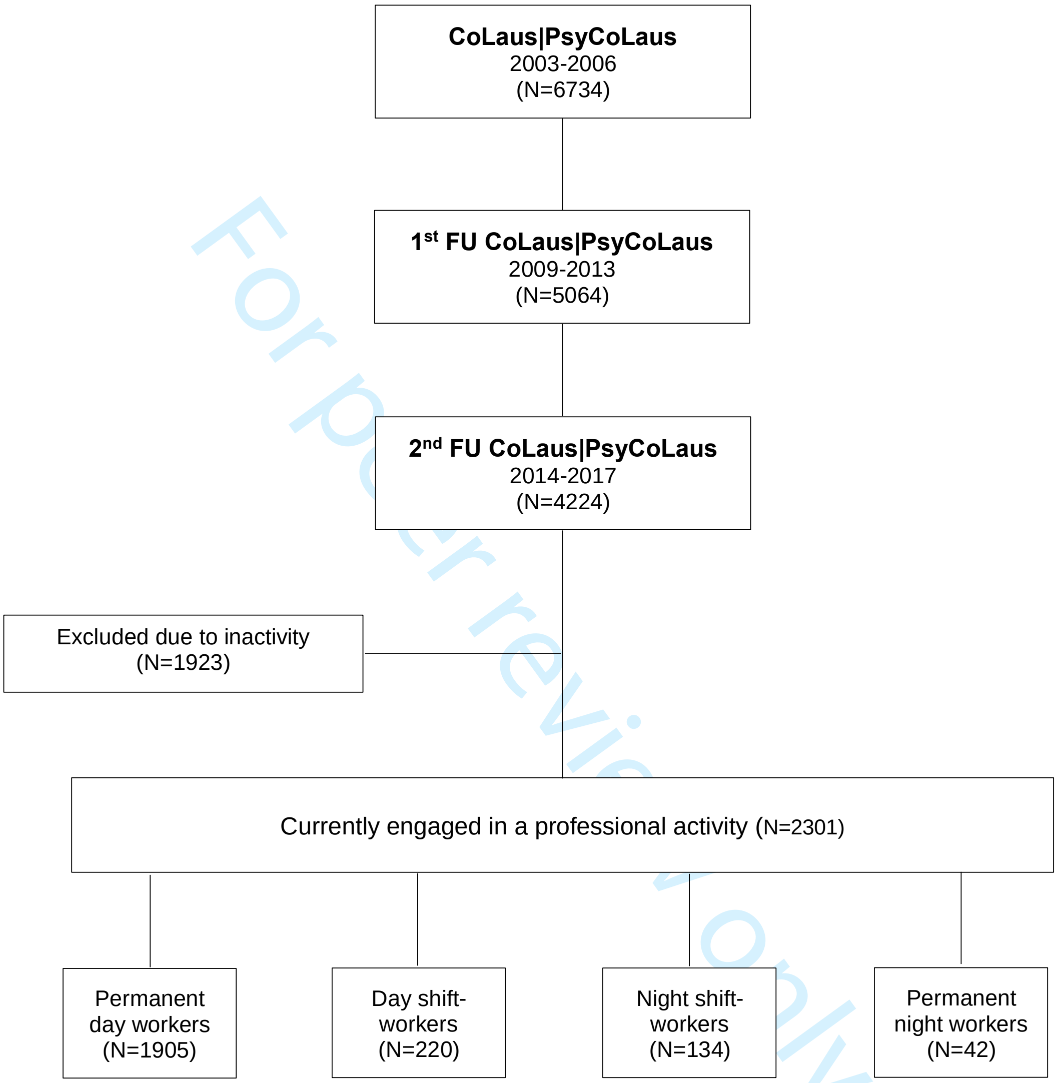

Supplementary Table 1. Association of metabolic syndrome with working schedule

	Crude		Model 1		Model 2	
	N (%)	*p*-value	OR (95% CI)	*p*-value	OR (95% CI)	*p*-value
**Men**	*n* = 1153		*n* = 1059		*n* = 847	
Permanent day workers	Ref	-	Ref	-	Ref	-
Day shift-workers	0.36 (0.18-0.74)	**0.005**	0.33 (0.15-0.69)	**0.004**	0.38 (0.17-0.87)	**0.022**
Night shift-workers	0.78 (0.45-1.34)	0.365	0.71 (0.40-1.26)	0.238	0.67 (0.33-1.36)	0.266
Permanent night workers	6.48 (2.40-17.46)	**<0.001**	6.00 (2.14-16.80)	**0.001**	4.37 (1.33-14.38)	**0.015**
**Women**	*n* = 1148		*n* = 1048		*n* = 798	
Permanent day workers	Ref	-	Ref	-	Ref	-
Day shift-workers	1.17 (0.66-2.05)	0.594	1.15 (0.62-2.12)	0.653	0.47 (0.17-1.24)	0.929
Night shift-workers	1.76 (0.80-3.89)	0.159	1.96 (0.86-4.46)	0.107	1.38 (0.42-4.57)	0.596
Permanent night workers	2.20 (0.81-6.03)	0.124	1.84 (0.60-5.64)	0.289	1.43 (0.28-7.19)	0.668

p-values <0.05 are in bold.

Model 1 was adjusted for age (continuous), age square (continuous) and educational level (middle, low, high). Model 2 was additionally adjusted for weekly alcohol consumption (continuous), smoking status (never, former, current) and daily total energy expenditure.

For peer review only

STROBE Statement—checklist of items that should be included in reports of observational studies

	Item No	Recommendation	Page number
**Title and abstract**	1	(*a*) Indicate the study's design with a commonly used term in the title or the abstract	1, 4
		(*b*) Provide in the abstract an informative and balanced summary of what was done and what was found	4
**Introduction**			
Background/rationale	2	Explain the scientific background and rationale for the investigation being reported	5, 6
Objectives	3	State specific objectives, including any prespecified hypotheses	6
**Methods**			
Study design	4	Present key elements of study design early in the paper	6, 7
Setting	5	Describe the setting, locations, and relevant dates, including periods of recruitment, exposure, follow-up, and data collection	6, 7
Participants	6	(*a*) *Cohort study*—Give the eligibility criteria, and the sources and methods of selection of participants. Describe methods of follow-up<br>*Case-control study*—Give the eligibility criteria, and the sources and methods of case ascertainment and control selection. Give the rationale for the choice of cases and controls<br>*Cross-sectional study*—Give the eligibility criteria, and the sources and methods of selection of participants	6, 7
		(*b*) *Cohort study*—For matched studies, give matching criteria and number of exposed and unexposed<br>*Case-control study*—For matched studies, give matching criteria and the number of controls per case	
Variables	7	Clearly define all outcomes, exposures, predictors, potential confounders, and effect modifiers. Give diagnostic criteria, if applicable	7, 8
Data sources/ measurement	8*	For each variable of interest, give sources of data and details of methods of assessment (measurement). Describe comparability of assessment methods if there is more than one group	*7, 8*
Bias	9	Describe any efforts to address potential sources of bias	9, 10
Study size	10	Explain how the study size was arrived at	7
Quantitative variables	11	Explain how quantitative variables were handled in the analyses. If applicable, describe which groupings were chosen and why	9, 10
Statistical methods	12	(*a*) Describe all statistical methods, including those used to control for confounding	9, 10, 11
		(*b*) Describe any methods used to examine subgroups and interactions	10
		(*c*) Explain how missing data were addressed	10
		(*d*) *Cohort study*—If applicable, explain how loss to follow-up was addressed<br>*Case-control study*—If applicable, explain how matching of cases and controls was addressed<br>*Cross-sectional study*—If applicable, describe analytical methods taking account of sampling strategy	
		(*e*) Describe any sensitivity analyses	

Continued on next page

For peer review only - http://bmjopen.bmj.com/site/about/guidelines.xhtml

**Results**			
Participants	13*	(a) Report numbers of individuals at each stage of study—eg numbers potentially eligible, examined for eligibility, confirmed eligible, included in the study, completing follow-up, and analysed	Fig. S1 10
		(b) Give reasons for non-participation at each stage	Fig. S1
		(c) Consider use of a flow diagram	Fig. S1
Descriptive data	14*	(a) Give characteristics of study participants (eg demographic, clinical, social) and information on exposures and potential confounders	11
		(b) Indicate number of participants with missing data for each variable of interest	Table 1
		(c) *Cohort study*—Summarise follow-up time (eg, average and total amount)	
Outcome data	15*	*Cohort study*—Report numbers of outcome events or summary measures over time	
		Case-control study—Report numbers in each exposure category, or summary measures of exposure	
		Cross-sectional study—Report numbers of outcome events or summary measures	*11, 12*
Main results	16	(*a*) Give unadjusted estimates and, if applicable, confounder-adjusted estimates and their precision (eg, 95% confidence interval). Make clear which confounders were adjusted for and why they were included	12, Fig. 1, Table S1
		(*b*) Report category boundaries when continuous variables were categorized	
		(*c*) If relevant, consider translating estimates of relative risk into absolute risk for a meaningful time period	
Other analyses	17	Report other analyses done—eg analyses of subgroups and interactions, and sensitivity analyses	12, 13, Table 3
**Discussion**			
Key results	18	Summarise key results with reference to study objectives	13
Limitations	19	Discuss limitations of the study, taking into account sources of potential bias or imprecision. Discuss both direction and magnitude of any potential bias	16
Interpretation	20	Give a cautious overall interpretation of results considering objectives, limitations, multiplicity of analyses, results from similar studies, and other relevant evidence	16, 17
Generalisability	21	Discuss the generalisability (external validity) of the study results	16, 17
**Other information**			
Funding	22	Give the source of funding and the role of the funders for the present study and, if applicable, for the original study on which the present article is based	18

*Give information separately for cases and controls in case-control studies and, if applicable, for exposed and unexposed groups in cohort and cross-sectional studies.

Note: An Explanation and Elaboration article discusses each checklist item and gives methodological background and published examples of transparent reporting. The STROBE checklist is best used in conjunction with this article (freely

For peer review only

available on the Web sites of PLoS Medicine at http://www.plosmedicine.org/, Annals of Internal Medicine at http://www.annals.org/, and Epidemiology at http://www.epidem.com/). Information on the STROBE Initiative is available at www.strobe-statement.org.

For peer review only

BMJ Open

Impact of night and shift work on metabolic syndrome and its components: A cross-sectional study in an active middle-to-older-aged population-based sample

Journal:	*BMJ Open*
Manuscript ID	bmjopen-2021-053591.R2
Article Type:	Original research
Date Submitted by the Author:	07-Dec-2021
Complete List of Authors:	Bayon, Virginie; CHUV) and University of Lausanne<br>Berger, Mathieu; CHUV, Center for Investigation and Research in Sleep<br>Solelhac, Geoffroy; CHUV) and University of Lausanne<br>Haba-Rubio, José; CHUV) and University of Lausanne<br>Marques-Vidal, Pedro; CHUV) and University of Lausanne, Department of Medicine, Internal Medicine<br>Strippoli, Marie-Pierre; CHUV) and University of Lausanne, Department of Psychiatry<br>Preisig, Martin; CHUV) and University of Lausanne, Department of Psychiatry<br>Leger, Damien; Centre du sommeil et de la vigilance, Hôtel Dieu, APHP; Université Paris Descartes, Sorbonne paris Cité, EA 7330 VIFASOM, Sommeil-Vigilance-Fatigue et Santé Publique,<br>Heinzer, Raphael; CHUV) and University of Lausanne
**Primary Subject Heading**:	Epidemiology
Secondary Subject Heading:	Diabetes and endocrinology, Occupational and environmental medicine
Keywords:	General endocrinology < DIABETES & ENDOCRINOLOGY, EPIDEMIOLOGY, Diabetes & endocrinology < INTERNAL MEDICINE

SCHOLARONE™
Manuscripts

I, the Submitting Author has the right to grant and does grant on behalf of all authors of the Work (as defined in the below author licence), an exclusive licence and/or a non-exclusive licence for contributions from authors who are: i) UK Crown employees; ii) where BMJ has agreed a CC-BY licence shall apply, and/or iii) in accordance with the terms applicable for US Federal Government officers or employees acting as part of their official duties; on a worldwide, perpetual, irrevocable, royalty-free basis to BMJ Publishing Group Ltd ("BMJ") its licensees and where the relevant Journal is co-owned by BMJ to the co-owners of the Journal, to publish the Work in this journal and any other BMJ products and to exploit all rights, as set out in our licence.

The Submitting Author accepts and understands that any supply made under these terms is made by BMJ to the Submitting Author unless you are acting as an employee on behalf of your employer or a postgraduate student of an affiliated institution which is paying any applicable article publishing charge ("APC") for Open Access articles. Where the Submitting Author wishes to make the Work available on an Open Access basis (and intends to pay the relevant APC), the terms of reuse of such Open Access shall be governed by a Creative Commons licence – details of these licences and which Creative Commons licence will apply to this Work are set out in our licence referred to above.

Other than as permitted in any relevant BMJ Author's Self Archiving Policies, I confirm this Work has not been accepted for publication elsewhere, is not being considered for publication elsewhere and does not duplicate material already published. I confirm all authors consent to publication of this Work and authorise the granting of this licence.

For peer review only

Impact of night and shift work on metabolic syndrome and its components: A cross-sectional study in an active middle-to-older-aged population-based sample

Short title: Impact of night and shift work

Virginie Bayon, MD[1,*] Mathieu Berger,PhD[1,*] Geoffroy Solelhac,MD[1] José Haba-Rubio,MD[1] Pedro Marques-Vidal,MD[2] Marie-Pierre F. Strippoli,MSc[3] Martin Preisig,MD[3] Damien Leger,MD[4,5] Raphael Heinzer,MD[1]

[1]Center for Investigation and Research in Sleep (CIRS), Lausanne University Hospital (CHUV) and University of Lausanne, Lausanne, Switzerland; [2]Department of Medicine, Internal Medicine, Lausanne University Hospital (CHUV) and University of Lausanne, Lausanne, Switzerland; [3]Department of Psychiatry, Lausanne University Hospital (CHUV) and University of Lausanne, Lausanne, Switzerland; [4]Université de Paris, VIFASOM (Equipe d'accueil 5330 VIgilance FAtigue SOMmeil et Santé Publique), Paris, France ; [5]APHP, Hôtel Dieu, Centre du Sommeil et de la Vigilance, Paris, France

[*]these authors contributed equally to this work

Corresponding Author: Mathieu Berger, PhD, Center for Investigation and Research in Sleep (CIRS), Lausanne University Hospital (CHUV), Rue du Bugnon 46, 1011 Lausanne, Switzerland. Tel: +41 21 314 86 45. E-mail: *mathieuberger@outlook.com*

For peer review only - http://bmjopen.bmj.com/site/about/guidelines.xhtml

Total word count (main text only): 3508

Number of Tables/Figures: 4/1

For peer review only

For peer review only - http://bmjopen.bmj.com/site/about/guidelines.xhtml

ABSTRACT

Objectives To examine the effects of work schedules on metabolic syndrome and its components in active middle-to-older-aged workers.

Methods A cross-sectional analysis including middle-to-older-aged active workers from the population-based CoLaus|PsyCoLaus study (Lausanne, Switzerland) was performed. Work schedule was self-reported and defined as follows: permanent day, day shift, night shift, and permanent night work. Associations between work schedule and the risk of metabolic syndrome and its components were analyzed using multivariable-adjusted logistic regressions.

Results A total of 2301 active workers (median age [interquartile range]: 55.4 [50.8-60.4], 50.1% women) were included. Of these, 1905 were permanent day workers, 220 were day shift workers, 134 were night shift workers and 42 were permanent night shift workers. There were significant interactions between sex and work schedule for metabolic syndrome, high triglycerides and visceral obesity. Men but not women permanent night workers had a higher prevalence of metabolic syndrome than permanent day workers in multivariable-adjusted analyses (OR 4.45 [95% CI 1.36-14.56]). Analysis of metabolic syndrome subcomponents showed that the association between work schedule and metabolic syndrome in men was mainly driven by visceral obesity (OR 3.35 [95% CI 1.04-10.76]). Conversely, women but not men working in night shift were at increased risk of having high triglycerides compared with permanent day workers (OR 2.92 [95% CI 1.03-8.27]).

Conclusions The risk of metabolic syndrome is higher in men working in permanent night shift compared with permanent day work and this association could be mediated by visceral obesity.

Keywords work schedule, abdominal obesity, risk factors

For peer review only - http://bmjopen.bmj.com/site/about/guidelines.xhtml

STRENGHS AND LIMITATIONS OF THIS STUDY

- This study evaluated the effects of work schedules on metabolic syndrome and its subcomponent in a middle-to-older-aged general population setting with a precise and extensive assessment of cardio-metabolic phenotypes.
- The association between different shift work schedules and metabolic syndrome was assessed after adjustment for multiple cofounders.
- Because the primary aim of the cohort was not to evaluate the impact of shift work, no precise characterization of workstations and work rhythms (hourly amplitude, direction of rotation, duration of rotations, and duration of exposition) was performed.
- A “healthy worker effect” with a selection of “night shift tolerant” workers cannot be ruled out given the older age of our sample.

For peer review only

Introduction

Due to economic constraints, efficiency needs or performance objectives, night and shift work (3x8) has become highly prevalent in modern societies. Approximately 18% of all European workers work shifts, and this rate is as high as 35% in some countries[1]. Non-standard working schedules (e.g. shift work, night work) are no longer limited to health and safety workers, but are spread across all industries and services, from manufacturing, to transport, telecommunications and more.

Night and shift work interfere with the physiological circadian rhythm, desynchronizing the biological clock, which can favor systemic inflammation[2]. Night and shift work are also associated with reduced and disturbed sleep[3]. Hence, both circadian disruption and short or poor sleep could be mediators explaining the relationship between night or shift work and chronic health conditions, including increased risk of cardiovascular and metabolic disorders[4]. Moreover, several laboratory-controlled studies showed that circadian rhythm desynchronization and sleep restriction have detrimental effects on neuroendocrine, inflammatory and immune functions[5].

The health-related impact of atypical work schedules has thus been a topic of interest for some time[6]. Sleep disturbances, decreased vigilance and increased risk of accidents are among the recognized short-term negative effects of night and shift work[7]. Longer-term health effects have also been described, and include increased risk of cardiovascular and metabolic disorders[8 9]. However, the impact of shift work on metabolic syndrome is not yet completely understood, particularly in the middle-to-older-aged population of workers though it is well established that the cardiometabolic risk gradually increases with advancing age.

For peer review only - http://bmjopen.bmj.com/site/about/guidelines.xhtml

Metabolic syndrome combines several interrelated metabolic risk factors associated with all-cause mortality[10]. Subjects with metabolic syndrome have a higher risk of cardiovascular disease mortality and morbidity[11]. Metabolic syndrome definition is based on five components: high blood pressure (BP), hyperglycemia, high triglycerides, low high-density lipoprotein (HDL) cholesterol and visceral obesity. A higher prevalence of metabolic syndrome and its components among night and shift workers has previously been suggested in some studies[12 13]. However, the specific effect of shift work and permanent night work remains largely unknown. Moreover, a recent systematic review concluded that there was insufficient evidence regarding the association between shift work and metabolic syndrome when confounding variables are taken into account[14].

Thus, using data of active middle-to-older aged workers from a population-based study, the aim of the present paper was to assess the cross-sectional association between metabolic syndrome and its components according to four types of work schedules (permanent day, day shift, night shift and permanent night shift work).

Methods

Study design cross-sectional analysis of a population-based cohort study.

Population CoLaus|PsyCoLaus is a population-based cohort exploring the biological, genetic, and environmental determinants of cardiovascular risk factors, cardiovascular diseases, and mental disorders in the middle-to-older-aged population of Lausanne, Switzerland. The methodological aspects (participant recruitment and follow-up) have been previously reported[15]. Briefly, a simple, non-stratified, random sample of 6,734 subjects from the Lausanne population aged 35-75 years was

For peer review only - http://bmjopen.bmj.com/site/about/guidelines.xhtml

recruited between 2003 and 2006. The baseline and three follow-up evaluations included physical and psychiatric exams, blood sampling, and self-completed questionnaires. All data analyzed in the present paper were obtained from the second physical follow-up evaluation (n = 4881), which took place between 2014 and 2017. The study was approved by the Institutional Ethics Committee of the University of Lausanne (decision reference 33/09) and written inform consent was obtained from all subjects.

Patient and Public Involvement

No patients or public were involved in this study design, conduct or analysis.

Exposure and eligibility criteria Professional activity and working hours were self-reported using the following questions: “Are you currently engaged in a professional activity?”; “What is your usual work schedule?” (day exclusively, rotation with no night work, rotation with night work, night work only). The number of work hours per week was also recorded. Participants not currently engaged in a professional activity were excluded from the present analysis. No other exclusion criteria were applied.

Outcome assessment Metabolic syndrome was defined according to the Joint Interim Statement [16] as the presence of at least three of the following five conditions: high BP (systolic BP ≥130 mm Hg or diastolic BP ≥85 mm Hg or use of antihypertensive medication); visceral obesity (waist circumference ≥88 cm in women or ≥102 cm in men); high triglycerides (≥1.7 mmol/L, or use of fibrates or nicotinic acid); low HDL-cholesterol levels (<1.30 mmol/L in women or <1.03 mmol/L in men, or use of fibrates or nicotinic acid); and high fasting plasma glucose (≥5.6 mmol/L or use of anti-diabetic medication). Blood pressure was measured three times on the

For peer review only - http://bmjopen.bmj.com/site/about/guidelines.xhtml

left arm using an Omron® HEM-907 (Matsusaka, Japan) automated oscillometric sphygmomanometer after at least a 10-min rest in the seated position. The mean of the last two measures was used. Venous blood samples were drawn after an overnight fast to measure the levels of glucose, HDL cholesterol, low HDL-cholesterol, and triglycerides. Biological assays were performed at the clinical laboratory of the Lausanne university hospital within two hours of blood collection. Index of insulin resistance during fasting was assessed by the homeostatic model assessment of insulin resistance (HOMA-IR), calculated as the fasting insulin level (in milliunits per milliliter) times the fasting glucose level (in milligrams per liter) divided by 405. Waist circumference was measured twice with a non-stretchable tape over the unclothed abdomen at the mid-point between the lowest rib and the iliac crest. Hip circumference was also measured twice at the greater trochanters. For waist and hip, the mean of the two measurements was used and the waist-to-hip ratio (WHR) was calculated.

Covariates The current socio-professional category was self-reported by participants. Sociodemographic (age, sex) and lifestyle (smoking habit, alcohol intake, coffee consumption) data were collected by self-administered questionnaires. Educational level was categorized as *low* (primary), *middle* (apprenticeship or secondary school) or *high* (university). Smoking status was categorized as *never, former* or *current*. Body weight and height were measured with participants standing without shoes in light indoor clothing. Body weight was measured in kilograms to the nearest 0.1 kg using a Seca™ scale (Seca, Hamburg, Germany). Height was measured to the nearest 5 mm using a Seca™ height gauge (Seca, Hamburg,

For peer review only - http://bmjopen.bmj.com/site/about/guidelines.xhtml

Germany). Body mass index (BMI) was defined as weight (kg)/height^2 (m^2). Obesity was defined as BMI ≥ 30 kg/m^2.

Medication use was coded according to the World Health Organization Anatomical Therapeutic Chemical (ATC) Classification System (http://www.whocc.no/atcddd). Drugs influencing sleep included hypnotics or sedatives (N05C), anxiolytics (N05B) and antipsychotics (N05A). Diabetes was defined as fasting plasma glucose levels ≥7.0 mmol/L or use of antidiabetic medication [17]. Hypertension was defined as systolic BP ≥140 mm Hg and/or diastolic BP ≥90 mm Hg, and/or current use of antihypertensive medication.

The presence of a current major depressive disorder was retrospectively assigned according to Diagnostic and Statistical Manual of Mental Disorders, Fourth Edition (DSM-IV) criteria with information collected at the second and third psychiatric follow-up evaluation using the French translation of the semi-structured Diagnostic Interview for Genetic Studies (DIGS). Cardiovascular disease was defined as previous stroke, heart attack, coronary artery bypass grafting or percutaneous coronary intervention.

Subjective sleep characteristics were determined using the Pittsburgh Sleep Quality Index (PSQI)[18], the Epworth Sleepiness Scale (ESS)[19], and the Berlin questionnaire for sleep-disordered breathing (SDB)[20]. Sleep quality was assessed with the PSQI and dichotomized into good/poor sleep quality (score ≤5/>5), and excessive daytime sleepiness (EDS) was defined as an ESS score >10). A Berlin score ≥2 was defined as indicating a high risk of SDB.

Dietary intake was evaluated using a validated Food Frequency Questionnaire (FFQ) querying the consumption of 97 different food items including portion size over

For peer review only - http://bmjopen.bmj.com/site/about/guidelines.xhtml

the previous 4 weeks. The daily total energy intake was obtained as well as the proportion of macronutrients, alcohol and fibers.

Physical activity was evaluated with the physical activity frequency questionnaire (PAFQ)[21]. The questionnaire lists 70 types of physical activity from various domains (e.g. occupational, housework, leisure time, sports, etc.) and participants indicated the number of days in the past week (0–7) and the duration per day (0–10 h, in 15-minute increments) for each activity. Energy expenditure corresponds to the sum of all the energy expenditure over one week divided by 7 to obtain a mean energy expenditure over a 24-hour period. Sedentary status was defined as spending more than 90% of daily energy in activities below moderate and high intensity (defined as requiring at least 4 times the basal metabolic rate [BMR]). The percentage of total energy >4 metabolic equivalents (METS) was also calculated to quantify moderate and high intensity physical activity.

Statistical analysis Data distribution was graphically assessed using a normal Q-Q plot. Data were presented as number of participants (%) for categorical variables, mean ± SD for normal distribution, or median and interquartile range for non-normally distributed continuous variables. Univariate analyses of continuous data were performed using one-way ANOVA or Kruskal Wallis test follow by Bonferroni's post-hoc or Tamhane's T2 as appropriate. Categorical variables were analyzed using Chi-square test or Fisher’s exact test as appropriate. The associations between working schedules (permanent day, day shift work, night shift work and permanent night work) and metabolic syndrome (and its subcomponents) were determined using logistic regression analysis. Prior to this, the interaction of sex with the metabolic syndrome and each of its subcomponents was tested. In case of significant

For peer review only - http://bmjopen.bmj.com/site/about/guidelines.xhtml

interaction, results were presented for both men and women, otherwise results were shown for the whole sample. Each cardiometabolic risk factor was first tested in univariate analysis (crude) then in two models with serial adjustment for potential confounders. Model 1 was adjusted for age (continuous), educational level (low, middle, high) and sex (except in case of significant sex*outcome interaction). Model 2: Model 1 plus weekly alcohol consumption (continuous), smoking status (never, former, current) and BMI (normal weight, overweight, obese; except for visceral obesity). Model 3: Model 2 plus daily total energy expenditure (continuous). Box-Tidwell tests were used to check the assumption of linearity for the logit of each covariate. If the assumption was violated, the square of the covariate was used or the covariate was transformed into categorical variable. To assess collinearity between covariates, a linear regression analysis including all covariates was performed, and the variance inflation factor (VIF) was calculated. A VIF ≤5 was considered as absence of multi-collinearity. Results from logistic regression are presented as OR values with 95% CI. Permanent day workers were considered as the reference group.

All statistical analyses were performed using IBM SPSS Statistics version 26.0 for Macintosh (IBM Corp). Significant results were considered for a two-sided test with $p < 0.05$.

Results

Population characteristics A total of 2301 participants were engaged in a professional activity at the second follow-up of the CoLaus|PsyCoLaus study. Among them, 1905 worked exclusively during the daytime (permanent day workers), 220 were rotation workers with no night work (day shift workers), 134 were rotation

For peer review only - http://bmjopen.bmj.com/site/about/guidelines.xhtml

workers with night work (night shift workers) and 42 worked exclusively during the night (permanent night workers) (Supplementary Figure 1).

Table 1 and 2 show the baseline characteristics of the sample according to the four different work schedules. The mean age of the participants was 56.2 ± 6.9 years and half of the sample (50.1%) were women. The proportions of men/women differed significantly according to work schedule: women were more likely to work in day shift and permanent night shift roles, while men were more likely to do night shift work. Mean BMI and waist circumference were significantly higher in night shift workers and permanent night workers compared with permanent day workers and day shift workers ($p<0.001$). Permanent night shift workers were more likely to smoke than other groups, whereas night shift workers were less sedentary than their counterparts. Lipid levels and blood glucose analysis, and sleep parameters in the different work schedule groups are also shown in Table 1 and 2.

Prevalence of metabolic syndrome and its components according to work schedules There were significant interactions between sex and work schedule for metabolic syndrome ($p=0.009$), high triglycerides ($p=0.043$) and visceral obesity ($p=0.047$), but not for high BP, high glucose and low HDL-cholesterol.

The prevalence of the metabolic syndrome was almost three times higher in men permanent night workers compared with men permanent day workers; a similar trend was found for the prevalence of visceral obesity and low HDL-cholesterol (Table 3). The prevalence of high glucose level in night shift workers and permanent night workers was nearly double that in permanent day workers (Table 3).

For peer review only - http://bmjopen.bmj.com/site/about/guidelines.xhtml

Association between metabolic syndrome and work schedules by patient sex

Compared to men permanent day workers, permanent night workers showed a higher risk of metabolic syndrome in univariate analysis (OR 6.48 [95% CI 2.40-17.46]; Supplementary Table 1). This significant association persisted after adjustment for age, educational level, alcohol consumption, smoking status and daily total energy expenditure (OR 4.45 [95% CI 1.36-14.56]) (Figure 1). Conversely, the risk of metabolic syndrome in day shift-workers was lower than that in permanent day workers in crude analysis (OR 0.36 [95% CI 0.18-0.74]), and after adjustment in models 1 and 2 and 3 (Supplementary Table 1). No significant association between work schedule and metabolic syndrome was found for women.

Association of each component of metabolic syndrome with work schedule

In men, the risk of visceral obesity in permanent night workers was significantly higher than that in permanent day workers, including after adjustment for covariates (Table 4). Moreover, the risk of elevated triglyceride levels in permanent night workers was increased in the crude analysis and after adjustment for age, educational level, alcohol consumption, smoking status and BMI (model 2), but was no longer significant in the fully adjusted model 3 (Table 4).

In women, night shift-workers showed a higher risk of elevated triglyceride levels, which persisted after multiple adjustments (Table 4).

Discussion

In our middle-to-older-aged active general population sample, we found differential associations between permanent night work and the risk of metabolic syndrome for men and women. Indeed, permanent night work was only associated with a higher

For peer review only - http://bmjopen.bmj.com/site/about/guidelines.xhtml

risk of metabolic syndrome in men but not in women. This association could be mediated by a higher risk of visceral obesity in men. The increased risk of metabolic syndrome is in line with previous studies[22]. Some studies even showed that the risk for the development of metabolic syndrome and each of its components gradually and independently increases with accumulated years of shift work[23]. Contrary to other studies, we found no association between permanent night work or night shift-work and metabolic syndrome in women[24 25]. In contrast to the findings on the metabolic syndrome as a whole, for the triglycerides component we found an increased risk of elevated concentrations among shift workers in women but not in men. This supports previous evidence from Karlsson *et al.* who also reported an elevated triglyceride level among shift workers in 60-year-old women[26].

While the mechanisms underlying the observed increased risk of metabolic syndrome in shift or night workers have not been fully elucidated, several explanatory hypotheses can be proposed. Firstly, sleep duration has been suggested to play a key role in the development of metabolic syndrome. A previous meta-analysis found that short sleep duration was significantly associated with a 27% increase in risk of metabolic syndrome whereas long sleep duration was not[27]. Similar results were found in both men and women. In our study, self-reported sleep duration did not differ between the different groups of workers and therefore does not explain the increased risk of metabolic syndrome observed in permanent night workers among men. However, we cannot rule out that our findings might have been different if objective sleep duration measures were used because objective and subjective sleep duration can differ significantly. Unfortunately, objective sleep assessment could not be included in our analysis. Moreover, sleep fragmentation or an alteration of sleep structure due to irregular sleep schedule or circadian rhythm misalignment in night

For peer review only - http://bmjopen.bmj.com/site/about/guidelines.xhtml

workers cannot be excluded and could be a possible explanation for the increased risk of metabolic syndrome[28 29].

Secondly, dietary habits could contribute to development of the metabolic syndrome in night or shift workers, but available studies on this subject are scarce. A cross-sectional study comparing 98 rotating shift workers to 100 regular day workers demonstrated that total energy intake and contributions of macronutrients did not differ between the two groups, except for saturated lipids (+10% in shift workers)[30]. However, meal distribution was different in the two groups. Similar to other studies[31 32], we failed to demonstrate a difference in food intake and macronutrients components between night shift workers or permanent night workers compared with permanent day workers. Available data from our study mean that, unfortunately, we cannot rule out the possibility that night shift workers may have had a different circadian distribution of food intake rather than an increase in total daily intake[33].

Thirdly, circadian rhythm desynchronization could be a major contributor to the increased risk of metabolic syndrome among night and shift workers. Still, the underlying pathophysiological mechanisms of this association remain poorly understood. Some animal studies suggested that reduced melatonin production, due to circadian rhythm disruption, could be associated with a higher rate of metabolic syndrome[34]. Furthermore, Fonken *et al.* hypothesized that exposure to light at night altered circadian organization and affected metabolic parameters in mice[35]. Their results emphasized that even weak night lighting (5 lux) is sufficient to desynchronize food consumption and physical activity rhythms, which could explain the observed metabolic disorders[34]. In humans, Corbalan-Tutau *et al.* reported a reduced daily amplitude in melatonin and cortisol circadian patterns associated with metabolic

For peer review only - http://bmjopen.bmj.com/site/about/guidelines.xhtml

disturbances in women[36]. Unfortunately, we did not measure melatonin and cortisol to confirm these findings in our sample.

With regard to physical activity, we surprisingly found that night shift workers were more active than day shift workers and permanent day workers. This may be due to greater opportunities to perform a physical activity compared with other diurnal workers or to more physically active work among night shift workers, although this should be interpreted with caution due to limited agreement between estimates of activity obtained by PAFQ and those obtained from accelerometers[37].

Finally, the higher risk of metabolic syndrome we observed in night shift workers may be explained by a vitamin D deficiency[38]. It has been shown that high levels of vitamin D among middle-aged and elderly populations are associated with a substantial decrease in cardiovascular disease, type 2 diabetes and metabolic syndrome[39]. Although we did not measure the vitamin D levels in our different groups of workers, we can hypothesize that permanent night workers have lower exposure to sunlight and may therefore be at higher risk of vitamin D deficiency[40].

In our study, among the components of the metabolic syndrome, an elevated risk of visceral obesity was found in men permanent night workers. This finding is consistent with a recent meta-analysis which found that shift workers had a higher frequency of abdominal obesity than other obesity types and permanent night workers demonstrated a 29% higher risk of central obesity than rotating shift workers[41].

The main strength of the present study is its large population-based sample of middle-to-older-aged workers with a precise and extensive assessment of cardio-metabolic phenotypes. Indeed, previous studies were mainly performed in younger specific populations of workers or in particular sectors of activity, such as public

For peer review only - http://bmjopen.bmj.com/site/about/guidelines.xhtml

health and emergency, which limit the generalizability to other types of shift or night work. In addition, most studies have assessed the risk of metabolic syndrome in shift workers compared with day workers, but few studies have differentiated between shift workers, permanent night workers and shift workers with and without night work.

There are also some limitations that need to be mentioned. First, this study had a cross-sectional design which did not allow to assess causality but only cross-sectional associations that remain to be confirmed in prospective studies. Because the primary aim of the CoLaus|PsyCoLaus study was not to evaluate the impact of shift work, the questions related to shift work were only asked at the follow-up 2 (2014-2017), preventing us to investigate longitudinal associations. Moreover, no precise characterization of workstations and work rhythms (hourly amplitude, direction of rotation, duration of rotations, and duration of exposition) was performed. Likewise, it would have been interesting to have any information regarding food intakes or other habits in the workplaces. Second, a “healthy worker effect” with a selection of “night shift tolerant” workers cannot be ruled out given the older age of our sample. Third, our sample of permanent night workers is rather small but we may assume that workers move away from night shift work with advancing age due to poorer tolerability and less family constraints. Fourth, there were some missing data on self-reported sleep habits and diet parameters and, despite the use of validated questionnaires, declaration bias remains possible. Similarly, only self-reported physical activity was assessed in this study and it would have been interesting to have objective measures of physical activity and sleep to more accurately investigate their influence.

CONCLUSION

For peer review only - http://bmjopen.bmj.com/site/about/guidelines.xhtml
For peer review only

Only men permanent night workers were at increased risk of metabolic syndrome compared with permanent day workers, and this association persisted after adjustment for sociodemographic confounders and daily total energy expenditure. From a clinical point of view, we advise monitor of not only BMI but also visceral obesity, particularly in men permanent night workers. Further prospective studies are needed to confirm theses cross-sectional results and elucidate the underline mechanisms.

For peer review only

For peer review only - http://bmjopen.bmj.com/site/about/guidelines.xhtml

ARTICLE INFORMATION

Acknowledgments

The authors thank Prof. Mehdi Tafti, Prof. Vincent Mooser, Daniela Andries and Nadia Tobback for their important contribution to the HypnoLaus and CoLaus|PsyCoLaus Cohorts, the Lausanne population who volunteered to participate in the CoLaus|PsyCoLaus and HypnoLaus studies, as well as the whole team of CoLaus|PsyCoLaus.

Funding

The HypnoLaus and the CoLaus/ PsyCoLaus studies were and are supported by research grants from GlaxoSmithKline ('not applicable'), the Faculty of Biology and Medicine of Lausanne ('not applicable'), the Swiss National Science Foundation (grants 3200B0-105993, 3200B0-118308, 33CSCO-122661, 33CS30-139468 and 33CS30- 148401), Leenaards Foundation ('not applicable'), and Vaud Pulmonary League ('not applicable'). The contents of this research are solely the responsibility of the authors.

Author Disclosures

The authors have no conflicts of interest to declare.

Contributors

VB, MB, PMV, MP and RH designed the study. JHR, PMV, MPFS, MP and RH collected the data. MB performed the statistical analysis. VB, MB, GS, JHR, PMV, MPFS, MP, DL and RH interpreted the data. VB and MB wrote the first draft of the manuscript and GS, JHR, PMV, MPFS, MP, DL and RH critically reviewed the

For peer review only

For peer review only - http://bmjopen.bmj.com/site/about/guidelines.xhtml

manuscript. All authors undertake to give final approval of the version to be published and agree to be accountable for all aspects of the work. VB is the guarantor of this work and, as such, had full access to all the data in the study and takes responsibility for the integrity of the data and the accuracy of the data analysis.

Data availability statement

Data may be obtained from a third party and are not publicly available. All data relevant to the study are included in the article or uploaded as supplementary information.

References

1 Nappo N. Is there an association between working conditions and health? An analysis of the Sixth European Working Conditions Survey data. *PLoS One* 2019;14:e0211294.

2 Puttonen S, Viitasalo K, Harma M. Effect of shiftwork on systemic markers of inflammation. *Chronobiol Int* 2011;28:528-35.

3 Sallinen M, Kecklund G. Shift work, sleep, and sleepiness - differences between shift schedules and systems. *Scand J Work Environ Health* 2010;36:121-33.

4 Knutson KL, Spiegel K, Penev P, Van Cauter E. The metabolic consequences of sleep deprivation. *Sleep Med Rev* 2007;11:163-78.

5 Faraut B, Bayon V, Leger D. Neuroendocrine, immune and oxidative stress in shift workers. *Sleep Med Rev* 2013;17:433-44.

6 Kecklund G, Axelsson J. Health consequences of shift work and insufficient sleep. *BMJ* 2016;355:i5210.

For peer review only - http://bmjopen.bmj.com/site/about/guidelines.xhtml

7 Drake CL, Roehrs T, Richardson G, Walsh JK, Roth T. Shift work sleep disorder: prevalence and consequences beyond that of symptomatic day workers. *Sleep* 2004;27:1453-62.

8 Morikawa Y, Nakagawa H, Miura K et al. Effect of shift work on body mass index and metabolic parameters. *Scand J Work Environ Health* 2007;33:45-50.

9 Wang D, Ruan W, Chen Z, Peng Y, Li W. Shift work and risk of cardiovascular disease morbidity and mortality: A dose-response meta-analysis of cohort studies. *Eur J Prev Cardiol* 2018;25:1293-1302.

10 Wu SH, Liu Z, Ho SC. Metabolic syndrome and all-cause mortality: a meta-analysis of prospective cohort studies. *Eur J Epidemiol* 2010;25:375-84.

11 Galassi A, Reynolds K, He J. Metabolic syndrome and risk of cardiovascular disease: a meta-analysis. *Am J Med* 2006;119:812-9.

12 Karlsson B, Knutsson A, Lindahl B. Is there an association between shift work and having a metabolic syndrome? Results from a population based study of 27,485 people. *Occup Environ Med* 2001;58:747-52.

13 Violanti JM, Burchfiel CM, Hartley TA et al. Atypical work hours and metabolic syndrome among police officers. *Arch Environ Occup Health* 2009;64:194-201.

14 Canuto R, Garcez AS, Olinto MT. Metabolic syndrome and shift work: a systematic review. *Sleep Med Rev* 2013;17:425-31.

15 Firmann M, Mayor V, Vidal PM et al. The CoLaus study: a population-based study to investigate the epidemiology and genetic determinants of cardiovascular risk factors and metabolic syndrome. *BMC Cardiovasc Disord* 2008;8:6.

16 Alberti KG, Eckel RH, Grundy SM et al. Harmonizing the metabolic syndrome: a joint interim statement of the International Diabetes Federation Task Force on Epidemiology and Prevention; National Heart, Lung, and Blood Institute; American

For peer review only

Heart Association; World Heart Federation; International Atherosclerosis Society; and International Association for the Study of Obesity. *Circulation* 2009;120:1640-5.

17 American Diabetes A. Diagnosis and classification of diabetes mellitus. *Diabetes Care* 2014;37 Suppl 1:S81-90.

18 Buysse DJ, Reynolds CF, 3rd, Monk TH, Berman SR, Kupfer DJ. The Pittsburgh Sleep Quality Index: a new instrument for psychiatric practice and research. *Psychiatry Res* 1989;28:193-213.

19 Johns MW. A new method for measuring daytime sleepiness: the Epworth sleepiness scale. *Sleep* 1991;14:540-5.

20 Netzer NC, Stoohs RA, Netzer CM, Clark K, Strohl KP. Using the Berlin Questionnaire to identify patients at risk for the sleep apnea syndrome. *Ann Intern Med* 1999;131:485-91.

21 Bernstein M, Sloutskis D, Kumanyika S, Sparti A, Schutz Y, Morabia A. Data-based approach for developing a physical activity frequency questionnaire. *Am J Epidemiol* 1998;147:147-54.

22 Wang F, Zhang L, Zhang Y et al. Meta-analysis on night shift work and risk of metabolic syndrome. *Obes Rev* 2014;15:709-20.

23 De Bacquer D, Van Risseghem M, Clays E, Kittel F, De Backer G, Braeckman L. Rotating shift work and the metabolic syndrome: a prospective study. *Int J Epidemiol* 2009;38:848-54.

24 Lim YC, Hoe VCW, Darus A, Bhoo-Pathy N. Association between night-shift work, sleep quality and metabolic syndrome. *Occup Environ Med* 2018;75:716-723.

25 Guo Y, Rong Y, Huang X et al. Shift work and the relationship with metabolic syndrome in Chinese aged workers. *PLoS One* 2015;10:e0120632.

For peer review only - http://bmjopen.bmj.com/site/about/guidelines.xhtml

For peer review only

26 Karlsson B, Knutsson A, Lindahl B. Is there an association between shift work and having a metabolic syndrome? Results from a population based study of 27,485 people. *Occupational and Environmental Medicine* 2001;58:747-752.

27 Xi B, He D, Zhang M, Xue J, Zhou D. Short sleep duration predicts risk of metabolic syndrome: a systematic review and meta-analysis. *Sleep Med Rev* 2014;18:293-7.

28 Stamatakis KA, Punjabi NM. Effects of sleep fragmentation on glucose metabolism in normal subjects. *Chest* 2010;137:95-101.

29 Tasali E, Leproult R, Ehrmann DA, Van Cauter E. Slow-wave sleep and the risk of type 2 diabetes in humans. *Proc Natl Acad Sci U S A* 2008;105:1044-9.

30 Esquirol Y, Bongard V, Mabile L, Jonnier B, Soulat JM, Perret B. Shift work and metabolic syndrome: respective impacts of job strain, physical activity, and dietary rhythms. *Chronobiol Int* 2009;26:544-59.

31 de Assis MA, Kupek E, Nahas MV, Bellisle F. Food intake and circadian rhythms in shift workers with a high workload. *Appetite* 2003;40:175-83.

32 Morikawa Y, Miura K, Sasaki S et al. Evaluation of the effects of shift work on nutrient intake: a cross-sectional study. *J Occup Health* 2008;50:270-8.

33 Molzof HE, Wirth MD, Burch JB et al. The impact of meal timing on cardiometabolic syndrome indicators in shift workers. *Chronobiol Int* 2017;34:337-348.

34 Fonken LK, Workman JL, Walton JC et al. Light at night increases body mass by shifting the time of food intake. *Proc Natl Acad Sci U S A* 2010;107:18664-9.

35 Fonken LK, Nelson RJ. The effects of light at night on circadian clocks and metabolism. *Endocr Rev* 2014;35:648-70.

For peer review only

For peer review only - http://bmjopen.bmj.com/site/about/guidelines.xhtml

36 Corbalan-Tutau D, Madrid JA, Nicolas F, Garaulet M. Daily profile in two circadian markers "melatonin and cortisol" and associations with metabolic syndrome components. *Physiol Behav* 2014;123:231-5.

37 Verhoog S, Gubelmann C, Guessous I, Bano A, Franco OH, Marques-Vidal P. Comparison of the Physical Activity Frequency Questionnaire (PAFQ) with accelerometry in a middle-aged and elderly population: The CoLaus study. *Maturitas* 2019;129:68-75.

38 Ju SY, Jeong HS, Kim DH. Blood vitamin D status and metabolic syndrome in the general adult population: a dose-response meta-analysis. *J Clin Endocrinol Metab* 2014;99:1053-63.

39 Parker J, Hashmi O, Dutton D et al. Levels of vitamin D and cardiometabolic disorders: systematic review and meta-analysis. *Maturitas* 2010;65:225-36.

40 Daugaard S, Garde AH, Hansen AM, Vistisen HT, Rejnmark L, Kolstad HA. Indoor, outdoor, and night work and blood concentrations of vitamin D and parathyroid hormone. *Scand J Work Environ Health* 2018;44:647-657.

41 Sun M, Feng W, Wang F et al. Meta-analysis on shift work and risks of specific obesity types. *Obes Rev* 2018;19:28-40.

For peer review only

Table 1. Baseline characteristics according to work schedules

	Permanent day workers (*n* = 1905)	Day shift workers (*n* = 220)	Night shift workers (*n* = 134)	Permanent night workers (*n* = 42)	*p*-value	N Total
**Demographics & anthropometrics**						
Age (years)	55.0 (50.0–60.0)	55.0 (50.5–59.5)	54.5 (50.4–58.6)	53.0 (48.8–57.2)	0.070	2275
Men, n (%)	958 (50.3)	89 (40.5) [a]	88 (65.7) [a]	18 (42.9)	**<0.001**	2301
Educational level, n (%)					**<0.001**	2300
Low	791 (41.5)	112 (50.9)	71 (53.0)	30 (71.4) [a]		
High	591 (31.0) [a]	43 (19.5)	23 (17.2)	2 (4.8)		
Body-mass index (kg/m^2)	25.4 (22.6–28.5)	25.5 (23.1–27.6)	26.0 (23.2–30.0) [b]	27.9 (25.4–31.3) [b,c]	**<0.001**	2228
Waist circumference (cm)	89.5 (81.0–98.5)	89.0 (81.4–96.0)	93.0 (84.3–102.0) [b,c]	95.0 (85.3–109.0) [b,c]	**<0.001**	2227
Waist to hip ratio	0.88 ± 0.09	0.87 ± 0.08	0.90 ± 0.09 [b,c]	0.90 ± 0.09	**0.013**	2227
**Risk factors**						
Metabolic syndrome, n (%)	327 (17.2)	25 (11.4)	25 (18.7)	17 (40.5)[a]	**<0.001**	2301
Number of metabolic risk factors[†]	1 (0–2)	1 (0–2)	1 (0–2)	2 (1–3) [b,c]	**0.006**	2301
Current major depressive disorder, n (%)	115 (7.9)	19 (11.7)	10 (9.6)	4 (12.1)	0.319	1756
Hypertension, n (%)	653 (34.9)	80 (36.7)	45 (33.6)	12 (28.6)	0.764	2263
Diabetes, n (%)	100 (5.4)	8 (3.7)	18 (13.6) [a]	4 (9.5)	**<0.001**	2231
Dyslipidemia, n (%)	413 (22.5)	51 (23.6)	29 (22.1)	16 (38.1)	0.123	2226
Sleep drugs, n (%)	109 (5.7)	14 (6.4)	4 (3.0)	4 (9.5)	0.367	2301
Cardiovascular disease, n (%)[††]	55 (2.9)	12 (5.5)	5 (3.7)	3 (7.1)	0.102	2291
**Risk factors**						
Smoking status, n (%)					**0.011**	2246
Former	689 (37.0)	86 (40.8)	57 (43.8)	9 (22.0) [a]		
Curent	404 (21.7)	44 (20.9)	22 (16.9)	18 (43.9) [a]		

For peer review only - http://bmjopen.bmj.com/site/about/guidelines.xhtml

For peer review only

Alcohol (units/week)	4 (1–9)	3 (0–7)	3 (0–7)	2 (0–6)	**0.010**	2162
Coffee consumption, n (%)					0.961	2222
None	186 (10.1)	23 (11.0)	13 (10.2)	4 (9.8)		
1-3 cups/day	1154 (62.6)	134 (64.1)	78 (60.9)	28 (68.3)		
≥4 cups/day	504 (27.3)	52 (24.9)	37 (28.9)	9 (22.0)		
Total energy intake (Kcals/day)	1756 ± 664	1761 ± 654	1828 ± 719	1853 ± 619	0.603	1996
Physical activity						
Total energy expenditure (Kcals/day)	2656 (2297–3076)	2698 (2336–3046)	3118 (2735–3578) [b,c]	2663 (2356–3164)	**<0.001**	1828
Activity ≥4 MET (% total activity)	10.1 (1.9–18.4)	8.8 (1.2–20.2)	14.4 (4.9–25.3) [b]	6.5 (0.3–16.1)	**0.005**	1828
Sedentary status, n (%)	758 (49.2)	92 (55.1)	32 (34.4) [a]	16 (57.1)	**0.011**	1828
**Blood analysis**						
Total cholesterol (mmol/L)	5.3 ± 0.9	5.4 ± 0.9	5.3 ± 0.9	5.4 ± 1.0	0.928	2226
HDL cholesterol (mmol/L)	1.5 (1.2–1.9)	1.6 (1.3–1.9)	1.4 (1.2–1.8)	1.4 (1.1–1.7)	**0.013**	2226
LDL cholesterol (mmol/L)	3.2 ± 0.8	3.2 ± 0.8	3.2 ± 0.8	3.2 ± 0.9	0.958	2226
Triglycerides (mmol/L)	1.0 (0.8–1.4)	1.0 (0.8–1.5)	1.1 (0.8–1.5)	1.2 (0.9–1.8)	0.278	2226
Fasting glucose (mmol/L)	5.2 (4.9–5.5)	5.1 (4.8–5.5)	5.2 (4.9–5.8)	5.5 (5–5.9)	**0.026**	2226
Insulin (microIU/mL)	7 (4.8–10.6)	7.2 (4.6–10.9)	7.3 (5.0–11.7)	8.8 (6.5–12.9)	**0.027**	2218
HOMA-IR[†††]	1.6 (1.1–2.6)	1.6 (1.0–2.6)	1.7 (1.2–3.2)	2.1 (1.5–3.4)	**0.012**	2218

Data are presented as mean ± SD or median and interquartile range for continuous variables and number of participants (%) for categorical variables. P-value < 0.05 are shown in bold. [a] adjusted residual >│ 2│; [b] statistically different from "day only"; [c] statistically different from "shift work without night". † Metabolic risk factor corresponded to the five risk factors which defined the metabolic syndrome according to the Joint Interim Statement [16]: Systolic blood pressure ≥130 mmHg or diastolic blood pressure ≥85 mmHg or use of antihypertensive medication; waist circumference ≥88 cm in women or ≥102 cm in men); triglycerides ≥1.7 mmol/L, or use of fibrates or nicotinic acid; HDL-cholesterol <1.30 mmol/L in women or <1.03 mmol/L in men, or use of fibrates or nicotinic acid; and high fasting plasma glucose (≥5.6 mmol/L or use of anti-diabetic

For peer review only - http://bmjopen.bmj.com/site/about/guidelines.xhtml
For peer review only

medication). †† Cardiovascular disease was defined by previous stroke, heart attack, coronary artery bypass grafting or percutaneous coronary intervention. ††† Index of insulin resistance during fasting was assessed by the homeostatic model assessment of insulin resistance (HOMA-IR), calculated as the fasting insulin level (in milliunits per milliliter) times the fasting glucose level (in milligrams per liter) divided by 405.

HDL: high density lipoprotein; LDL: low density lipoprotein; MET: metabolic equivalent of task.

For peer review only

Table 2. Working and sleep characteristics according to work schedules

	Permanent day workers (*n* = 1905)	Day shift workers (*n* = 220)	Night shift workers (*n* = 134)	Permanent night workers (*n* = 42)	*p*-value	N Total
**Working characteristics**						
Number of working hours/week	38.0 ± 14.7	38.7 ± 15.2	43.1 ± 18.1	38.0 ± 15.2	0.260	2285
Work time, n (%)					0.397	2258
Full-time	1569 (83.8)	181 (84.6)	111 (86.0)	39 (92.9)		
<50%	304 (16.2)	33 (15.4)	18 (14.0)	3 (7.1)		
Example of physical intensity at work, n (%)					**<0.001**	2135
Sedentary (sitting/driving)	1409 (79.5)	105 (51.2)	66 (55.0)	14 (37.8)		
Pushing wheelbarrow	283 (16.0)	81 (39.5)	40 (33.3)	16 (43.2)		
Unloading a truck without assist.	81 (4.6)	19 (9.3)	14 (11.7)	7 (18.9)		
**Sleep & vigilance**						
Epworth Sleepiness Scale score	6 (4–8)	5 (3–8)	6 (4–9)	5 (3–8)	0.623	1786
Excessive daytime sleepiness, n (%)‡	182 (12.1)	20 (12.6)	14 (14.0)	3 (11.1)	0.950	1786
Poor sleep quality, n (%)‡‡	415 (31.5)	46 (37.4)	27 (32.1)	7 (35.0)	0.600	1542
High risk of SDB, n (%)§	321 (21.3)	34 (21.0)	29 (28.4)	8 (27.6)	0.323	1800
Self-reported total sleep time (h)	6.9 ± 1.0	6.8 ± 0.9	6.9 ± 1.0	7.1 ± 1.3	0.507	1542

Data are presented as mean ± SD or median and interquartile range for continuous variables and number of participants (%) for categorical variables. P-value < 0.05 are shown in bold. ‡ Excessive daytime sleepiness was defined by an Epworth Sleepiness Scale score >10; ‡‡ Poor sleep quality was defined by a Pittsburgh Sleep Quality Index score >5; § High risk of SDB was defined by a Berlin score >2.

For peer review only - http://bmjopen.bmj.com/site/about/guidelines.xhtml

Table 3. Prevalence of metabolic syndrome and its subcomponents according to work schedule

	**Permanent day workers (*n* = 1905)**	**Day shift workers (*n* = 220)**	**Night shift workers (*n* = 134)**	**Permanent night workers (*n* = 42)**	***p*-value**
Metabolic syndrome					
Men	226 (23.6)	9 (10.1)	17 (19.3)	12 (66.7)	**<0.001**
Women	101 (10.7)	16 (12.2)	8 (17.4)	5 (20.8)	0.225
High BP	826 (43.4)	91 (41.4)	64 (47.8)	23 (54.8)	0.313
High glucose	472 (24.8)	50 (22.7)	47 (35.1)	16 (38.1)	**0.010**
High triglycerides					
Men	243 (26.2)	25 (29.1)	18 (21.2)	11 (61.1)	**0.006**
Women	86 (9.5)	16 (12.3)	9 (19.6)	3 (12.5)	0.183
Low HDL-cholesterol	201 (10.9)	19 (8.8)	10 (7.6)	9 (21.4)	0.064
Visceral obesity					
Men	220 (23.7)	16 (18.6)	23 (26.7)	11 (61.1)	**0.002**
Women	302 (33.3)	55 (42.3)	21 (45.7)	11 (45.8)	0.051

Data are presented as n (%).

Where there was an interaction of outcome*sex, results are presented separately for men and women, otherwise for the whole cohort.

For peer review only

For peer review only - http://bmjopen.bmj.com/site/about/guidelines.xhtml

Table 4. Association of each component of the metabolic syndrome with work schedule

	Crude		Model 1		Model 2		Model 3	
	**OR (95% CI)**	***p*-value**	**OR (95% CI)**	***p*-value**	**OR (95% CI)**	***p*-value**	**OR (95% CI)**	***p*-value**
**High BP**	*n* = 2301		*n* = 2274		*n = 2066*		*n* = 1731	
Day shift-workers	0.92 (0.69-1.22)	0.572	1.05 (0.78-1.42)	0.746	1.02 (0.73-1.42)	0.907	1.06 (0.73-1.54)	0.757
Night shift-workers	1.19 (0.84-1.70)	0.321	1.02 (0.70-1.49)	0.912	0.92 (0.61-1.39)	0.682	1.01 (0.62-1.63)	0.983
Permanent night workers	1.58 (0.86-2.92)	0.144	1.78 (0.93-3.41)	0.081	1.60 (0.77-3.31)	0.204	1.90 (0.79-4.58)	0.155
**High fasting glucose**	*n* = 2301		*n* = 2274		*n = 2066*		*n* = 1731	
Day shift-workers	0.89 (0.64-1.25)	0.504	1.05 (0.74-1.50)	0.776	1.07 (0.73-1.58)	0.735	1.04 (0.66-1.63)	0.883
Night shift-workers	1.64 (1.13-2.37)	**0.009**	1.36 (0.91-2.02)	0.135	1.44 (0.93-2.24)	0.106	1.26 (0.74-2.14)	0.389
Permanent night workers	1.87 (0.99-3.51)	0.052	2.14 (1.07-4.29)	**0.031**	1.70 (0.79-3.64)	0.173	1.31 (0.52-3.29)	0.572
**High triglycerides**								
Men	*n* = 1117		*n* = 1116		*n* = 1038		*n* = 886	
Day shift-workers	1.16 (0.71-1.88)	0.562	1.14 (0.70-1.87)	0.593	1.30 (0.77-2.19)	0.324	1.32 (0.73-2.40)	0.360
Night shift-workers	0.76 (0.44-1.30)	0.313	0.74 (0.43-1.28)	0.287	0.86 (0.49-1.52)	0.604	0.97 (0.52-1.84)	0.936
Permanent night workers	4.43 (1.70-11.56)	**0.002**	4.31 (1.64-11.30)	**0.003**	3.50 (1.19-10.26)	**0.023**	3.27 (0.99-10.77)	0.051
Women	*n* = 1109		*n* = 1105		*n* = 1020		*n* = 837	
Day shift-workers	1.34 (0.76-2.37)	0.309	1.34 (0.75-2.38)	0.320	1.19 (0.63-2.24)	0.594	0.92 (0.41-2.03)	0.828
Night shift-workers	2.33 (1.09-4.99)	**0.030**	2.29 (1.06-4.95)	**0.035**	2.65 (1.14-6.15)	**0.023**	2.92 (1.03-8.27)	**0.044**
Permanent night workers	1.37 (0.40-4.68)	0.618	1.36 (0.39-4.73)	0.625	1.09 (0.30-3.97)	0.899	0.53 (0.06-4.32)	0.549
**Low HDL-cholesterol**	*n* = 2226		*n* = 2221		*n = 2058*		*n* = 1723	
Day shift-workers	0.79 (0.50-1.29)	0.336	0.75 (0.46-1.23)	0.255	0.62 (0.28-1.40)	0.252	0.74 (0.39-1.39)	0.348

For peer review only - http://bmjopen.bmj.com/site/about/guidelines.xhtml

Night shift-workers	0.67 (0.35-1.30)	0.240	0.60 (0.31-1.18)	0.138	0.58 (0.29-1.15)	0.116	0.66 (0.30-1.45)	0.300
Permanent night workers	2.22 (1.05-4.71)	**0.038**	1.90 (0.89-4.08)	0.099	1.61 (0.71-3.64)	0.252	1.47 (0.52-4.18)	0.468
**Visceral obesity**								
Men	*n* = 1119		*n* = 1118		*n* = 1043		*n* = 890	
Day shift-workers	0.74 (0.42-1.29)	0.288	0.75 (0.42-1.34)	0.333	0.84 (0.47-1.51)	0.561	0.72 (0.36-1.42)	0.341
Night shift-workers	1.18 (0.71-1.94)	0.525	1.11 (0.66-1.84)	0.704	1.06 (0.61-1.85)	0.257	0.84 (0.44-1.63)	0.612
Permanent night workers	5.06 (1.94-13.22)	**0.001**	5.27 (1.99-13.98)	**0.001**	4.79 (1.64-14.03)	**0.004**	3.35 (1.04-10.76)	**0.042**
Women	*n* = 1108		*n* = 1104		*n* = 1022		*n* = 839	
Day shift-workers	1.47 (1.01-2.14)	**0.043**	1.48 (1.01-2.17)	**0.043**	1.31 (0.87-1.97)	0.194	1.05 (0.65-1.70)	0.852
Night shift-workers	1.70 (0.93-3.06)	0.086	1.79 (0.98-3.29)	0.059	1.91 (1.01-3.62)	**0.047**	1.51 (0.66–3.10)	0.324
Permanent night workers	1.70 (0.75-3.84)	0.203	1.69 (0.73-3.92)	0.219	1.75 (0.72-4.23)	0.217	0.83 (0.23-2.99)	0.971

Data are presented as odds ratio (OR) and 95% confidence intervals (CI). For each component analyzed, the "permanent day workers" were considered as the reference group. *p*-values <0.05 are in bold. Model 1 was adjusted for age (continuous), age square (continuous), sex (except for sex subanalysis) and educational level (middle, low, high). Model 2 was additionally adjusted for weekly alcohol consumption (continuous), smoking status (never, former, current) and for BMI (normal weight, overweight, obese) (except for visceral obesity). Model 3 was additionally adjusted for daily total energy expenditure (continuous).

For peer review only - http://bmjopen.bmj.com/site/about/guidelines.xhtml

For peer review only

FIGURE LEGENDS

Figure 1. Multivariable-adjusted risk of metabolic syndrome according to work schedule and sex.

Data are presented on a logarithmic scale and were analyzed using multivariable logistic regression with adjustment for age, educational level, weekly alcohol consumption, smoking status and daily total energy expenditure (Model 3).

For peer review only

For peer review only - http://bmjopen.bmj.com/site/about/guidelines.xhtml

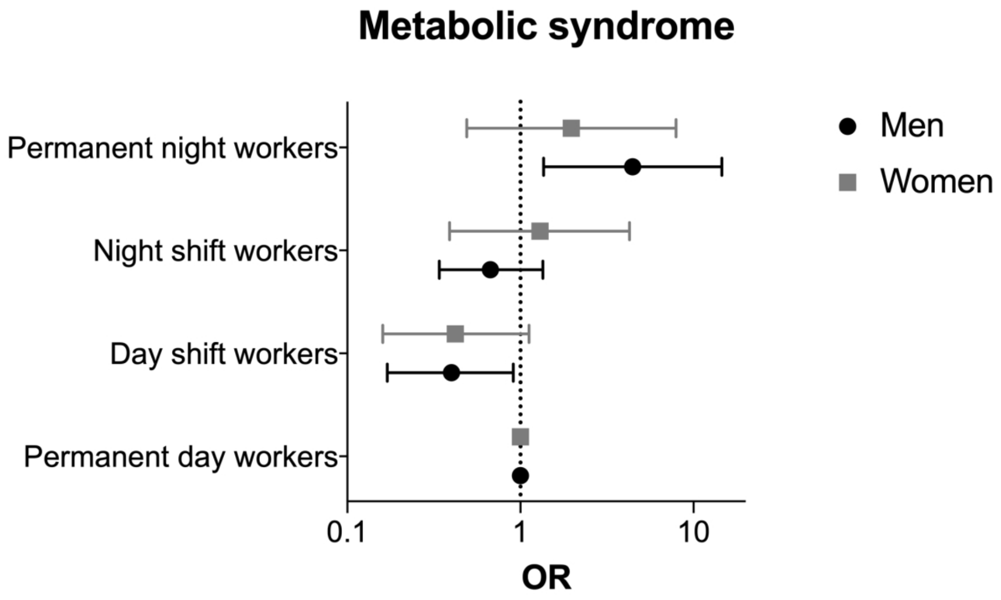

Figure 1. Multivariable-adjusted risk of metabolic syndrome according to work schedule and sex. Data are presented on a logarithmic scale and were analyzed using multivariable logistic regression with adjustment for age, educational level, weekly alcohol consumption, smoking status and daily total energy expenditure (Model 3).

177x109mm (300 x 300 DPI)

SUPPLEMENTAL MATERIAL

Impact of night and shift work on metabolic syndrome and its components in an active middle-aged population-based sample

Virginie Bayon,[1,*] Mathieu Berger,[1,*] Geoffroy Solelhac,[1] José Haba-Rubio,[1] Pedro Marques-Vidal,[2] Marie-Pierre Strippoli,[3] Martin Preisig,[3] Damien Leger,[4,5] Raphael Heinzer[1]

[1]Center for Investigation and Research in Sleep (CIRS), Lausanne University Hospital (CHUV) and University of Lausanne, Lausanne, Switzerland; [2]Department of Medicine, Internal Medicine, Lausanne University Hospital (CHUV) and University of Lausanne, Lausanne, Switzerland; [3]Department of Psychiatry, Lausanne University Hospital (CHUV) and University of Lausanne, Lausanne, Switzerland; [4]Université de Paris, VIFASOM (Equipe d'accueil 5330 VIgilance FAtigue SOMmeil et Santé Publique), Paris, France ; [5]APHP, Hôtel Dieu, Centre du Sommeil et de la Vigilance, Paris, France.

For peer review only

Online Data Supplement:

- **1 Supplementary Figure**
- **1 Supplementary Table**

Supplementary Figure 1. Study flowchart

FU: Follow-up

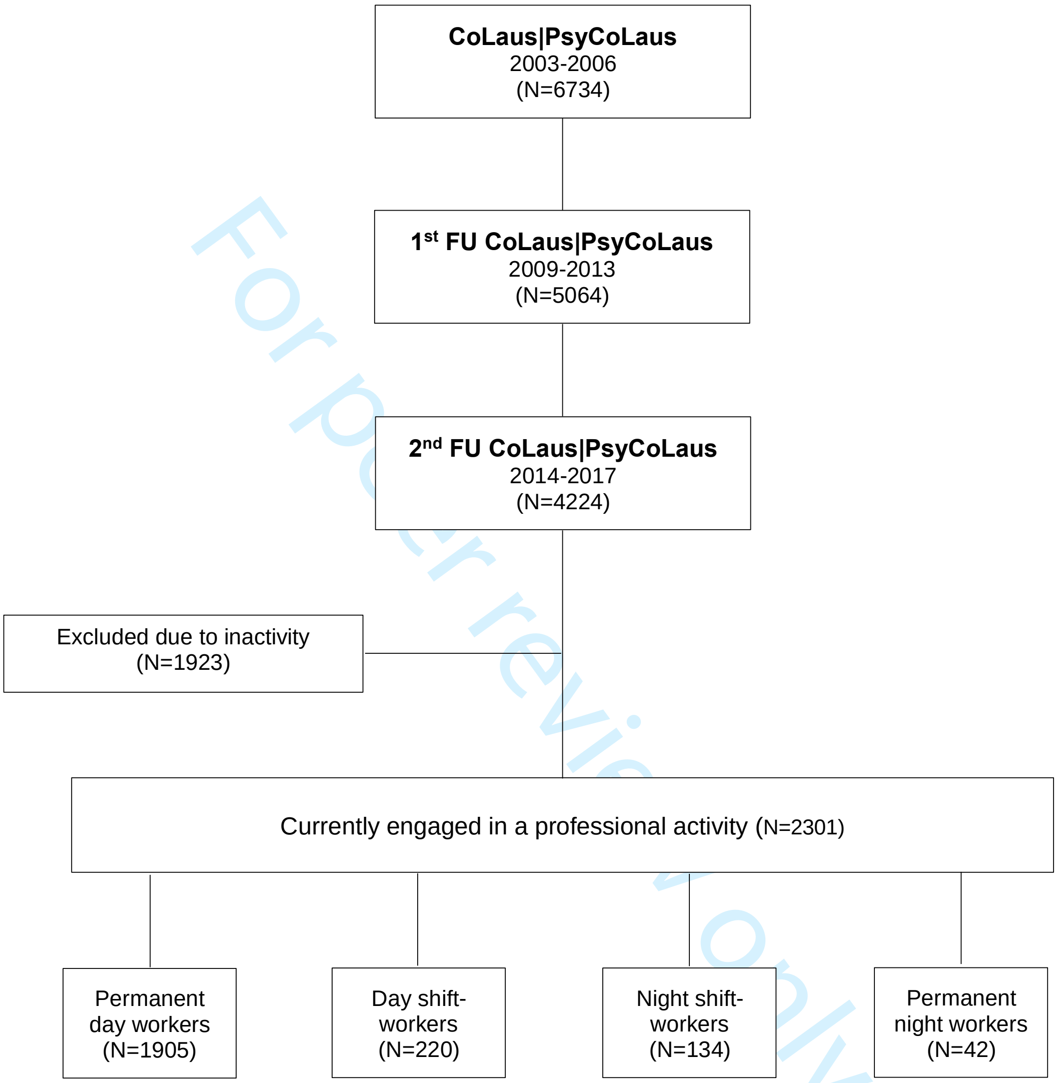

Supplementary Table 1. Association of metabolic syndrome with working schedule

	Crude		Model 1		Model 2	
	N (%)	*p*-value	OR (95% CI)	*p*-value	OR (95% CI)	*p*-value
**Men**	*n* = 1153		*n* = 1059		*n* = 847	
Permanent day workers	Ref	-	Ref	-	Ref	-
Day shift-workers	0.36 (0.18-0.74)	**0.005**	0.33 (0.15-0.69)	**0.004**	0.38 (0.17-0.87)	**0.022**
Night shift-workers	0.78 (0.45-1.34)	0.365	0.71 (0.40-1.26)	0.238	0.67 (0.33-1.36)	0.266
Permanent night workers	6.48 (2.40-17.46)	**<0.001**	6.00 (2.14-16.80)	**0.001**	4.37 (1.33-14.38)	**0.015**
**Women**	*n* = 1148		*n* = 1048		*n* = 798	
Permanent day workers	Ref	-	Ref	-	Ref	-
Day shift-workers	1.17 (0.66-2.05)	0.594	1.15 (0.62-2.12)	0.653	0.47 (0.17-1.24)	0.929
Night shift-workers	1.76 (0.80-3.89)	0.159	1.96 (0.86-4.46)	0.107	1.38 (0.42-4.57)	0.596
Permanent night workers	2.20 (0.81-6.03)	0.124	1.84 (0.60-5.64)	0.289	1.43 (0.28-7.19)	0.668

p-values <0.05 are in bold.

Model 1 was adjusted for age (continuous), age square (continuous) and educational level (middle, low, high). Model 2 was additionally adjusted for weekly alcohol consumption (continuous), smoking status (never, former, current) and daily total energy expenditure.

For peer review only

STROBE Statement—checklist of items that should be included in reports of observational studies

	Item No	Recommendation	Page number
**Title and abstract**	1	(*a*) Indicate the study's design with a commonly used term in the title or the abstract	1, 4
		(*b*) Provide in the abstract an informative and balanced summary of what was done and what was found	4
**Introduction**			
Background/rationale	2	Explain the scientific background and rationale for the investigation being reported	5, 6
Objectives	3	State specific objectives, including any prespecified hypotheses	6
**Methods**			
Study design	4	Present key elements of study design early in the paper	6, 7
Setting	5	Describe the setting, locations, and relevant dates, including periods of recruitment, exposure, follow-up, and data collection	6, 7
Participants	6	(*a*) *Cohort study*—Give the eligibility criteria, and the sources and methods of selection of participants. Describe methods of follow-up<br>*Case-control study*—Give the eligibility criteria, and the sources and methods of case ascertainment and control selection. Give the rationale for the choice of cases and controls<br>*Cross-sectional study*—Give the eligibility criteria, and the sources and methods of selection of participants	6, 7
		(*b*) *Cohort study*—For matched studies, give matching criteria and number of exposed and unexposed<br>*Case-control study*—For matched studies, give matching criteria and the number of controls per case	
Variables	7	Clearly define all outcomes, exposures, predictors, potential confounders, and effect modifiers. Give diagnostic criteria, if applicable	7, 8
Data sources/ measurement	8*	For each variable of interest, give sources of data and details of methods of assessment (measurement). Describe comparability of assessment methods if there is more than one group	*7, 8*
Bias	9	Describe any efforts to address potential sources of bias	9, 10
Study size	10	Explain how the study size was arrived at	7
Quantitative variables	11	Explain how quantitative variables were handled in the analyses. If applicable, describe which groupings were chosen and why	9, 10
Statistical methods	12	(*a*) Describe all statistical methods, including those used to control for confounding	9, 10, 11
		(*b*) Describe any methods used to examine subgroups and interactions	10
		(*c*) Explain how missing data were addressed	10
		(*d*) *Cohort study*—If applicable, explain how loss to follow-up was addressed<br>*Case-control study*—If applicable, explain how matching of cases and controls was addressed<br>*Cross-sectional study*—If applicable, describe analytical methods taking account of sampling strategy	
		(*e*) Describe any sensitivity analyses	

Continued on next page

For peer review only - http://bmjopen.bmj.com/site/about/guidelines.xhtml

**Results**			
Participants	13*	(a) Report numbers of individuals at each stage of study—eg numbers potentially eligible, examined for eligibility, confirmed eligible, included in the study, completing follow-up, and analysed	Fig. S1 10
		(b) Give reasons for non-participation at each stage	Fig. S1
		(c) Consider use of a flow diagram	Fig. S1
Descriptive data	14*	(a) Give characteristics of study participants (eg demographic, clinical, social) and information on exposures and potential confounders	11
		(b) Indicate number of participants with missing data for each variable of interest	Table 1
		(c) *Cohort study*—Summarise follow-up time (eg, average and total amount)	
Outcome data	15*	*Cohort study*—Report numbers of outcome events or summary measures over time	
		Case-control study—Report numbers in each exposure category, or summary measures of exposure	
		Cross-sectional study—Report numbers of outcome events or summary measures	*11, 12*
Main results	16	(*a*) Give unadjusted estimates and, if applicable, confounder-adjusted estimates and their precision (eg, 95% confidence interval). Make clear which confounders were adjusted for and why they were included	12, Fig. 1, Table S1
		(*b*) Report category boundaries when continuous variables were categorized	
		(*c*) If relevant, consider translating estimates of relative risk into absolute risk for a meaningful time period	
Other analyses	17	Report other analyses done—eg analyses of subgroups and interactions, and sensitivity analyses	12, 13, Table 3
**Discussion**			
Key results	18	Summarise key results with reference to study objectives	13
Limitations	19	Discuss limitations of the study, taking into account sources of potential bias or imprecision. Discuss both direction and magnitude of any potential bias	16
Interpretation	20	Give a cautious overall interpretation of results considering objectives, limitations, multiplicity of analyses, results from similar studies, and other relevant evidence	16, 17
Generalisability	21	Discuss the generalisability (external validity) of the study results	16, 17
**Other information**			
Funding	22	Give the source of funding and the role of the funders for the present study and, if applicable, for the original study on which the present article is based	18

*Give information separately for cases and controls in case-control studies and, if applicable, for exposed and unexposed groups in cohort and cross-sectional studies.

Note: An Explanation and Elaboration article discusses each checklist item and gives methodological background and published examples of transparent reporting. The STROBE checklist is best used in conjunction with this article (freely

For peer review only - http://bmjopen.bmj.com/site/about/guidelines.xhtml

available on the Web sites of PLoS Medicine at http://www.plosmedicine.org/, Annals of Internal Medicine at http://www.annals.org/, and Epidemiology at http://www.epidem.com/). Information on the STROBE Initiative is available at www.strobe-statement.org.

For peer review only

For peer review only - http://bmjopen.bmj.com/site/about/guidelines.xhtml
